# Correction: Transgenic Fatal Familial Insomnia Mice Indicate Prion Infectivity-Independent Mechanisms of Pathogenesis and Phenotypic Expression of Disease

**DOI:** 10.1371/journal.ppat.1005046

**Published:** 2015-07-06

**Authors:** The PLOS Pathogens Staff

## Notice of Republication

This article was republished on June 23, 2015, to correct errors in Tables 1 and 2 that were introduced during the typesetting process. The publisher apologizes for the errors. Please download this article again to view the correct version. The corrected article is provided here for reference.

## Supporting Information

S1 File Republished, corrected article. (PDF)
