# Supplementary figures and images for: Transgenic Fatal Familial Insomnia Mice Indicate Prion Infectivity-Independent Mechanisms of Pathogenesis and Phenotypic Expression of Disease
Source: PLoS Pathog. 2015 Apr 16;11(4):e1004796. doi: 10.1371/journal.ppat.1004796 (PMC4400166; doi:10.1371/journal.ppat.1004796)

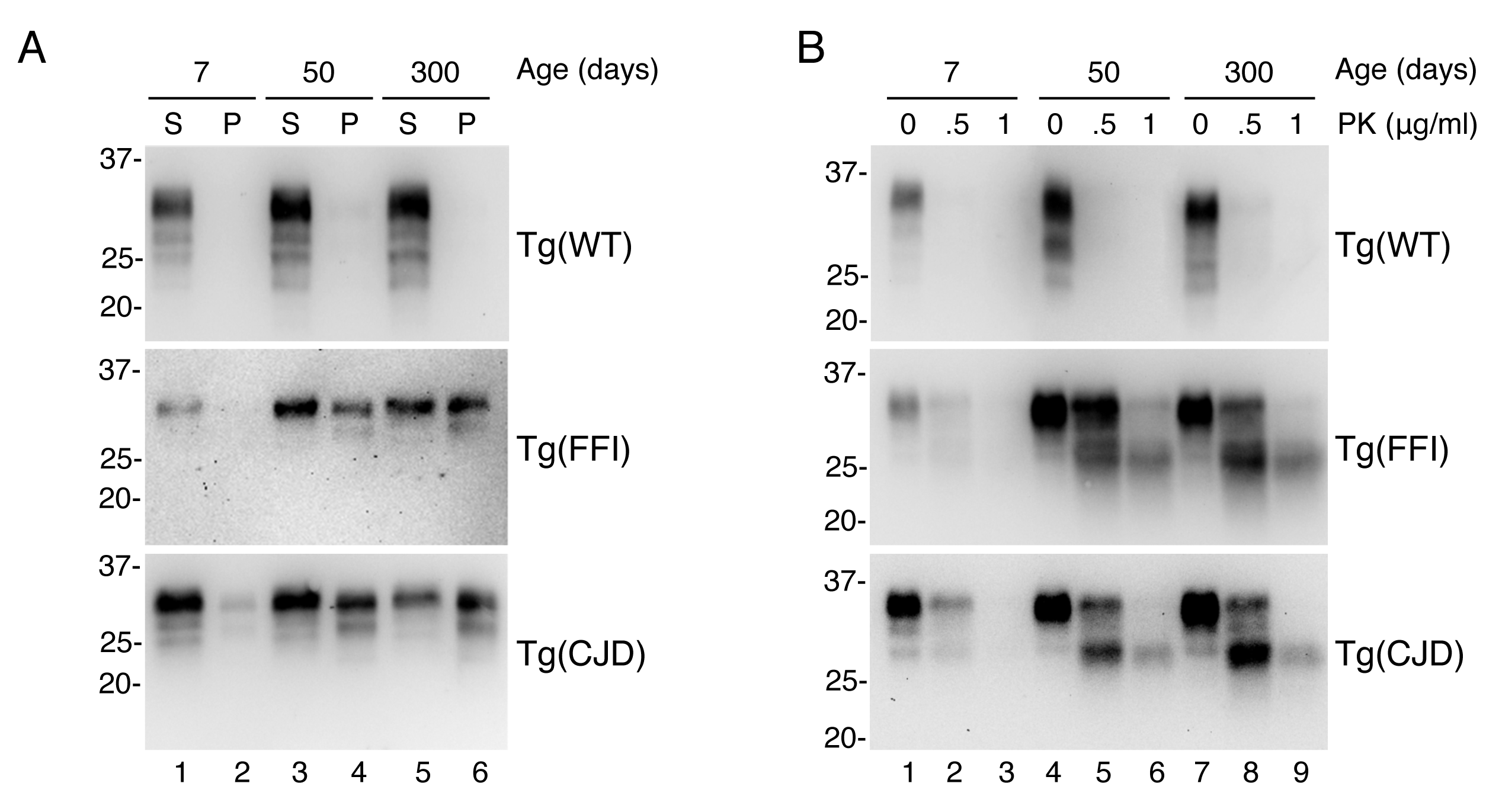

Supplement: S1 Fig — (A) Brain lysates from Tg(WT-E1+/-)/Prnp 0/0, Tg(FFI-26+/-)/Prnp 0/0 and Tg(CJD-66+/-)/Prnp 0/0 mice of the indicated ages were ultracentrifuged at 186,000 x g for 40 min, and PrP in the supernatants (S lanes) and pellets (P lanes) was analyzed by Western blotting using the 12B2 antibody. (B) Brain lysates were incubated with 0–2 μg of PK for 30 min at 37°C, and PrP was visualized by Western blotting using antibody 12B2. The undigested samples (0 μg/ml PK) represent 25 μg of protein, and the other samples 100 μg. (TIF) [file ppat.1004796.s001.tif]

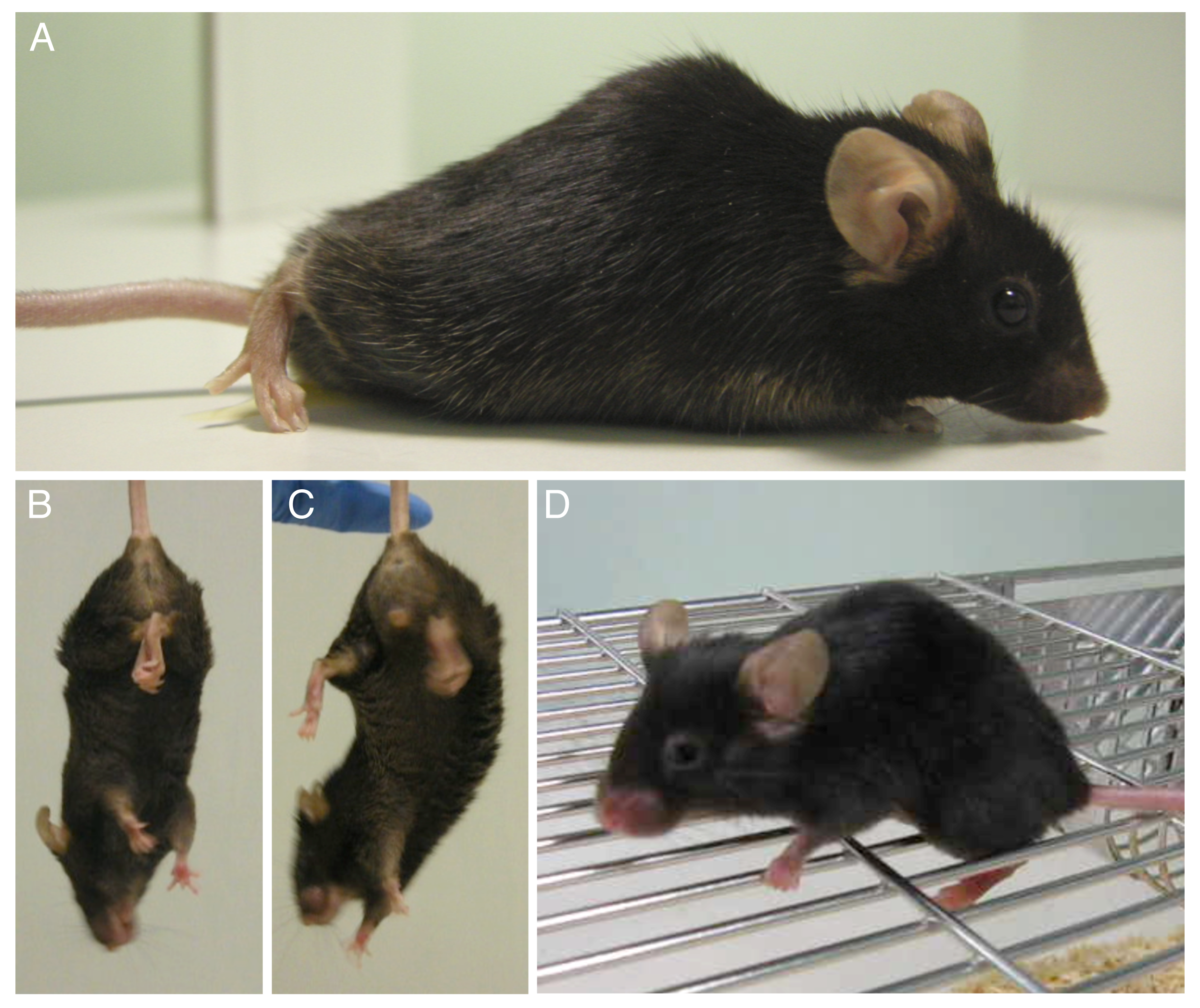

Supplement: S2 Fig — (A) The Tg(FFI-28+/-)/Prnp +/+ founder at 443 days of age shows kyphosis (hunchback position) and abnormal gait with extension of the hind limbs. (B) When suspended by its tail a Tg(FFI-10+/+)/Prnp 0/0 mouse aged 702 days tightly clasps its hind limbs, whereas a Tg(FFI-10+/-)/Prnp 0/0 littermate (C) splays its limbs. (D) A Tg(FFI-26+/-)/Prnp 0/0 mouse at 418 days is incapable of deambulating on a metal grill. (TIF) [file ppat.1004796.s002.tif]

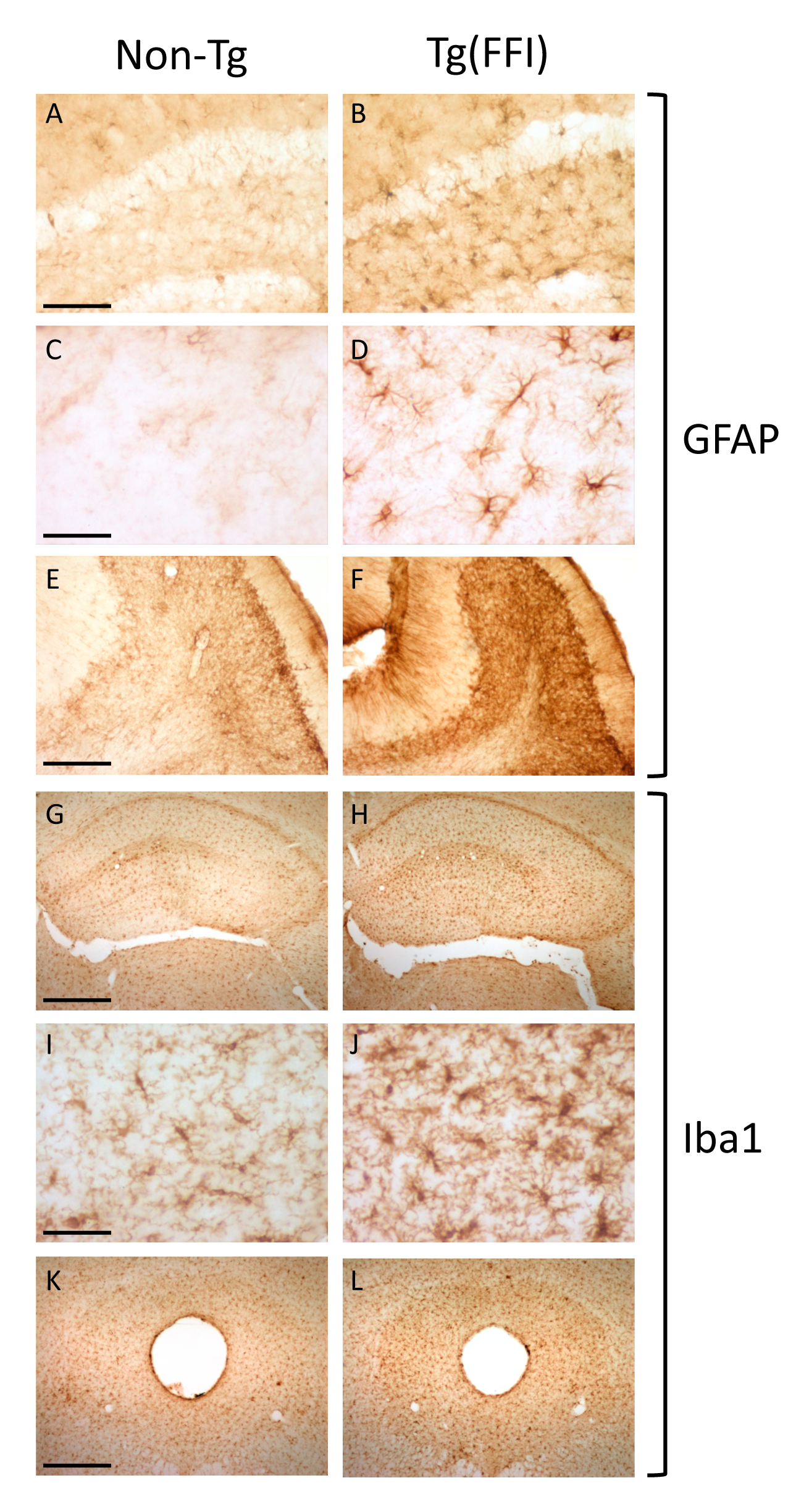

Supplement: S3 Fig — (A-F) Brain sections from Tg(FFI-26+/-)/Prnp 0/0 and non-Tg littermates aged 473 (A and B, hippocampus), 204 (C and D, cerebral cortex) and 198 (E and F, cerebellum) days, were stained with anti-glial fibrillar acidic protein (GFAP) antibody. Immunostaining revealed marked astrocytosis in Tg(FFI) but not in non-Tg mice. (G-L) Immunostaining with anti-ionized calcium binding adapter molecule 1 (Iba1) shows marked microgliosis in the hippocampus (G-J), and periaqueductal gray (K and L) of Tg(FFI-26+/-)/Prnp 0/0 mice at 533 (H and L) or 473 (J) days compared to non-Tg/Prnp 0/0 littermates. Results were similar with the anti-CD11b antibody. Scale bars = 100 μm in A and B, 50 μm in C, D, I and J, 200 μm in E and F, and 500 μm in G, H, K and L. (TIF) [file ppat.1004796.s003.tif]
