# Supplementary material for: Correction: Transgenic Fatal Familial Insomnia Mice Indicate Prion Infectivity-Independent Mechanisms of Pathogenesis and Phenotypic Expression of Disease
Source: PLoS Pathog. 2015 Jul 6;11(7):e1005046. doi: 10.1371/journal.ppat.1005046 (PMC4492966; doi:10.1371/journal.ppat.1005046)
Supplement: S1 File — (PDF) [file ppat.1005046.s001.pdf]

PLOS | PATHOGENS

RESEARCH ARTICLE

Transgenic Fatal Familial Insomnia Mice Indicate Prion Infectivity-Independent Mechanisms of Pathogenesis and Phenotypic Expression of Disease

Ihssane Bouybayoune[1‡], Susanna Mantovani[1¤a‡], Federico Del Gallo[2¤b‡], Ilaria Bertani[1], Elena Restelli[1], Liliana Comerio[1], Laura Tapella[1], Francesca Baracchi[2], Natalia Fernández-Borges[3], Michela Mangieri[4¤c], Cinzia Bisighini[5], Galina V. Beznoussenko[6], Alessandra Paladini[1], Claudia Balducci[1], Edoardo Micotti[1], Gianluigi Forloni[1], Joaquín Castilla[3,7], Fabio Fiordaliso[5], Fabrizio Tagliavini[4], Luca Imeri[2], Roberto Chiesa[1]*]

1 Department of Neuroscience, IRCCS—"Mario Negri" Institute for Pharmacological Research, Milan, Italy, **2** Department of Health Sciences, University of Milan Medical School, Milan, Italy, **3** CIC bioGUNE, Parque Tecnológico de Bizkaia, Derio, Spain, **4** Division of Neuropathology and Neurology, IRCCS Foundation "Carlo Besta" National Neurological Institute, Milan, Italy, **5** Bio-Imaging Unit, Department of Cardiovascular Research, IRCCS—"Mario Negri" Institute for Pharmacological Research, Milan, Italy, **6** IFOM—FIRC Institute of Molecular Oncology, Milan, Italy, **7** IKERBASQUE, Basque Foundation for Science, Bilbao, Spain

¤a Present address: School of Biomedical Sciences, The University of Queensland, Brisbane, Australia
¤b Present address: Department of Neurological and Movement Sciences, University of Verona, Verona, Italy
¤c Present address: Department of Neuropathology, Golgi Cenci Foundation, Abbiategrasso (MI), Italy
‡ These authors share first authorship on this work.
* roberto.chiesa@marionegri.it

Abstract

Fatal familial insomnia (FFI) and a genetic form of Creutzfeldt-Jakob disease (CJD[178]) are clinically different prion disorders linked to the D178N prion protein (PrP) mutation. The disease phenotype is determined by the 129 M/V polymorphism on the mutant allele, which is thought to influence D178N PrP misfolding, leading to the formation of distinctive prion strains with specific neurotoxic properties. However, the mechanism by which misfolded variants of mutant PrP cause different diseases is not known. We generated transgenic (Tg) mice expressing the mouse PrP homolog of the FFI mutation. These mice synthesize a misfolded form of mutant PrP in their brains and develop a neurological illness with severe sleep disruption, highly reminiscent of FFI and different from that of analogously generated Tg(CJD) mice modeling CJD[178]. No prion infectivity was detectable in Tg(FFI) and Tg(CJD) brains by bioassay or protein misfolding cyclic amplification, indicating that mutant PrP has disease-encoding properties that do not depend on its ability to propagate its misfolded conformation. Tg(FFI) and Tg(CJD) neurons have different patterns of intracellular PrP accumulation associated with distinct morphological abnormalities of the endoplasmic reticulum and Golgi, suggesting that mutation-specific alterations of secretory transport may contribute to the disease phenotype.

OPEN ACCESS

Citation: Bouybayoune I, Mantovani S, Del Gallo F, Bertani I, Restelli E, Comerio L, et al. (2015) Transgenic Fatal Familial Insomnia Mice Indicate Prion Infectivity-Independent Mechanisms of Pathogenesis and Phenotypic Expression of Disease. PLoS Pathog 11(4): e1004796. doi:10.1371/journal.ppat.1004796

Editor: Jason Bartz, Creighton University, UNITED STATES

Received: November 12, 2014

Accepted: March 9, 2015

Published: April 16, 2015

Copyright: © 2015 Bouybayoune et al. This is an open access article distributed under the terms of the Creative Commons Attribution License, which permits unrestricted use, distribution, and reproduction in any medium, provided the original author and source are credited.

Data Availability Statement: All relevant data are within the paper and its Supporting Information files.

Funding: This work was supported by grants from Telethon-Italy (TDRC00508TU and GGP12115; https://www.telethon.it/en) to RC, the Italian Ministry of Health (RF-INN-2008-1215065 and RF-2010-2314035; http://www.salute.gov.it/) to RC and FT, and Fondazione Cariplo (2010-0828 and 2012-0560; http://www.fondazionecariplo.it/en/index.html) to RC and FT. The funders had no role in study design, data

collection and analysis, decision to publish, or preparation of the manuscript.

Competing Interests: The authors have declared that no competing interests exist.

Author Summary

Genetic prion diseases are degenerative brain disorders caused by mutations in the gene encoding the prion protein (PrP). Different PrP mutations cause different diseases, including Creutzfeldt-Jakob disease (CJD) and fatal familial insomnia (FFI). The reason for this variability is not known, but assembly of the mutant PrPs into distinct aggregates that spread in the brain by promoting PrP aggregation may contribute to the disease phenotype. We previously generated transgenic mice modeling genetic CJD, clinically identified by dementia and motor abnormalities. We have now generated transgenic mice carrying the PrP mutation associated with FFI, and found that they develop severe sleep abnormalities and other key features of the human disorder. Thus, transgenic mice recapitulate the phenotypic differences seen in humans. The mutant PrPs in FFI and CJD mice are aggregated but unable to promote PrP aggregation. They accumulate in different intracellular compartments and cause distinct morphological abnormalities of transport organelles. These results indicate that mutant PrP has disease-encoding properties that are independent of its ability to self-propagate, and suggest that the phenotypic heterogeneity may be due to different effects of aggregated PrP on intracellular transport. Our study provides new insights into the mechanisms of selective neuronal dysfunction due to protein aggregation.

Introduction

Prion strains with unique self-templating and neurotoxic properties are thought to emerge spontaneously in humans carrying genetic prion disease-associated PrP mutations, dictating the phenotypic expression of disease. Here we report that transgenic (Tg) mice carrying the PrP mutation associated with one of these diseases (fatal familial insomnia, FFI) develop severe sleep disorders and other key phenotypic features of the human disease, different from those seen in analogously generated Tg mice expressing another prion disease-associated mutation (Creutzfeldt-Jakob disease, CJD). No prion infectivity is spontaneously generated in these mice, indicating that mutant PrP has disease-encoding properties that do not depend on self-templating competence.

Prion diseases are progressive and invariably fatal degenerative disorders of the central nervous system (CNS) that affect humans and other animals [1]. CJD, FFI and Gerstmann-Sträussler-Scheinker (GSS) syndrome are the most common forms in humans; scrapie of the goat and sheep, bovine spongiform encephalopathy, and chronic wasting disease of deer and elk are the best-known prion zoonoses [2]. Neuronal loss, gliosis, spongiform change (vacuolation of the neuropil in the gray matter) and in some cases amyloid deposits are typical neuropathological findings in prion diseases, which in humans usually present with loss of motor coordination and other motor abnormalities, dementia and neurophysiological deficits.

Similarly to other progressive neurodegenerative disorders, such as Alzheimer's disease (AD) and Parkinson's disease (PD), frontotemporal dementia and the tauopathies, prion diseases can arise sporadically or be genetically inherited; however, they can also be acquired by infection [3, 4]. The infectious agent (prion) is scrapie prion protein (PrP^{Sc}) [5]. This is a conformationally altered and aggregated isoform of the cellular prion protein (PrP^{C}) which propagates by imprinting its aberrant conformation onto native PrP^{C} molecules [6].

Genetic prion diseases are linked to point mutations and insertions in the *PRNP* gene encoding PrP^{C} on chromosome 20 [7]. Point mutations are mainly clustered in the protein's C terminus, leading to amino acid substitutions or protein truncations. The insertions consist of

additional copies of an octapeptide repeat in the N-terminal region, which normally contains one nonapeptide and four octapeptides. Mutant PrP is thought to misfold and aggregate spontaneously, eventually acquiring the PrP^{Sc} structure.

Different *PRNP* mutations are associated with distinct clinical and neuropathological phenotypes: CJD, FFI, GSS, PrP-cerebral amyloid angiopathy [7] and a recently described PrP systemic amyloidosis [8]. The disease phenotype is also influenced by *PRNP* polymorphic codon 129, where either methionine (M) or valine (V) can be encoded. A typical example is the prion disease linked to the substitution of aspartic acid (D) to asparagine (N) at codon 178, which, depending on the aminoacid encoded at polymorphic site 129, segregates with either FFI (D178N/M129), primarily characterized by severe sleep disorders and autonomic dysfunction, or CJD^{178} (D178N/V129), clinically identified by global cortical dementia and motor abnormalities [9]. The reason for this variability is not known. There is evidence that D178N/M129 and D178N/V129 PrPs differ in their folding and supramolecular assembly [10–12], but how conformational variants of the PrP polypeptide produce different diseases is not clear.

Only recently have we begun to understand how mutant PrP causes neurological dysfunction. PG14, a mouse (mo) PrP carrying a nine-octapeptide repeat insertion associated with GSS [13], and moPrP D177N/V128, homologous to the human CJD^{178} mutation, are partially retained in the neuronal endoplasmic reticulum (ER) [14, 15]. Intracellular accumulation of these mutants impairs the secretory transport of the voltage-gated calcium channel (VGCC) $\alpha_2\delta$-1 subunit, resulting in inefficient targeting of the VGCC complex to presynaptic terminals. This leads to inefficient glutamatergic neurotransmission in cerebellar granule neurons (CGNs) and abnormal motor behavior in Tg mice [15]. Thus in mouse models of GSS and CJD^{178}, ER retention of mutant PrP causes motor disease by altering the secretory trafficking of calcium channels essential for synaptic activity.

To further explore the mechanisms of mutant PrP neurotoxicity and, specifically, the role of the 129 polymorphism in directing the disease phenotype, we developed a mouse model of FFI. Here we describe Tg(FFI) mice expressing moPrP D177N/M128, which presented abnormalities in sleep-wake patterns and other pathological features highly reminiscent of FFI. Neurons in Tg(FFI) mice accumulate mutant PrP in the Golgi and show morphological alterations of this transport organelle. This suggests that different mutant PrPs may have different effects on secretory transport, potentially inducing specific functional abnormalities in neurons, hence clinically defined neurological diseases.

Results

Generation of Tg(FFI) Mice and Characterization of Mutant PrP

We produced Tg mice expressing moPrP D177N/M128 with or without an epitope tag for monoclonal antibody 3F4. We identified ten founders (four with and six without the 3F4 epitope). To generate the transgenic lines, referred to as Tg(FFI), founders were bred with PrP knockout mice ($Prnp^{0/0}$), so that the progeny expressed only mutant PrP. We established five Tg lines: one expressing 3F4-tagged (FFI-K5) and four untagged mutant PrP (FFI-10, FFI-15, FFI-26 and FFI-31). Transgene copy number and mutant PrP expression are shown in Table 1 and Fig 1A and 1B. Western blot analysis showed that unglycosylated PrP was under-represented (Fig 1), like in humans carrying the D178N mutation [16].

Mutant PrP in the mouse brain was largely insoluble (seen in pellet fractions in Fig 1C and 1D), and weakly protease-resistant (Fig 1E, lanes 5–8). After deglycosylation with PNGaseF, the PK-resistant fragment had an apparent molecular mass of 19 kDa (Fig 1F, lane 2), consistent with observations in FFI patients [16]. The PK-resistant fragment of D177N/V128 PrP in Tg(CJD) mice was also 19 kDa (Fig 1F, lane 4), different from mutant PrP from CJD^{178}

Table 1. Characteristics of founders carrying the D177N/M128 transgene.

Founder	3F4 epitope	Transgene copy number[a]	Tg PrP protein[b]
FFI-A6[c]	+	17	0.5X
FFI-K2[c]	+	3	0.01X
FFI-K5	+	18	0.7X
FFI-10	-	15	1X
FFI-15	-	2	0.5X
FFI-17[d]	-	ND	4X
FFI-26	-	31	2X
FFI-28[e]	-	ND	5X
FFI-31	-	1	0.03X

[a] Determined by quantitative PCR.

[b] Relative to endogenous PrP expression in non-Tg mice (data are for mice that are hemizygous for the transgene array).

[c] Founders euthanized at > 800 days of age, with no clinical symptoms.

[d] Founder died at 109 days of age because of lymphoma.

[e] Founder died with neurological symptoms at 477 days of age. ND, not determined.

doi:10.1371/journal.ppat.1004796.t001

patients, which has a PK-resistant core of 21 kDa [16], confirming our previous observations [14]. Detergent-insoluble and PK-resistant PrP was already detectable in 50 days old mice (S1 Fig), well before they developed clinical disease (see below).

Like in FFI Patients, Circadian Organization, Architecture, EEG Features and Amount of Sleep Are Deranged in Tg(FFI) Mice

As disruption of sleep is a key feature of FFI, we analyzed the sleep-wake patterns in Tg(FFI) mice. We used Tg(FFI-26)/*Prnp*$^{0/0}$ mice, which express the mutant protein at approximately twice the wild-type (WT) level (Table 1 and Fig 1B) and develop a fatal neurological syndrome with motor and cognitive deficits (see below). To assess the effect of co-expression of WT PrP, we also analyzed Tg(FFI-26)/*Prnp*$^{+/0}$ mice, in which one *Prnp* allele was reintroduced by back-crossing Tg(FFI-26)/*Prnp*$^{0/0}$ with C57BL/6J mice (hereafter referred to as non-Tg/*Prnp*$^{+/+}$).

Circadian organization of sleep and motor activity was lost in Tg(FFI)/*Prnp*$^{0/0}$ mice. As expected in nocturnal animals, non-Tg/*Prnp*$^{+/+}$ mice slept (summing up NREM and REM sleep) about twice as long during the day than during the night (2.1 ± 0.2 times). This was also true for non-Tg/*Prnp*$^{0/0}$, and Tg(FFI)/*Prnp*$^{+/0}$ mice, which slept 2.0 ± 0.1 and 2.4 ± 0.3 times more during the day than during the night, respectively. In contrast, Tg(FFI)/*Prnp*$^{0/0}$ mice slept only fifty percent more (1.5 ± 0.1 times; $p < 0.05$ by one-way ANOVA, $F_{3,31} = 5.672$).

The disorganization of sleep circadian rhythms in Tg(FFI)/*Prnp*$^{0/0}$ mice was confirmed by analysis of gross body movements. Non-Tg/*Prnp*$^{+/+}$, non-Tg/*Prnp*$^{0/0}$ and Tg(FFI)/*Prnp*$^{+/0}$ mice moved almost three to almost four times more during the night than during the day, respectively (2.9 ± 0.3, 3.9 ± 0.4 and 3.7 ± 0.6 times). Tg(FFI)/*Prnp*$^{0/0}$ mice moved only 1.6 ± 0.2 times more during the night than during the day ($p < 0.001$ by one-way ANOVA, $F_{3,31} = 10.819$).

Sleep continuity and organization were affected in Tg(FFI)/*Prnp*$^{0/0}$ mice. The number of transitions between different behavioral states (an indicator of broken sleep) was greater in Tg(FFI)/*Prnp*$^{0/0}$ mice than in non-Tg/*Prnp*$^{+/+}$ and non-Tg/*Prnp*$^{0/0}$ mice, during both the light and dark phases (Fig 2). In Tg(FFI)/*Prnp*$^{+/0}$ mice there were more transitions only in comparison to non-Tg/*Prnp*$^{+/+}$ mice, and only during the light phase (Fig 2).

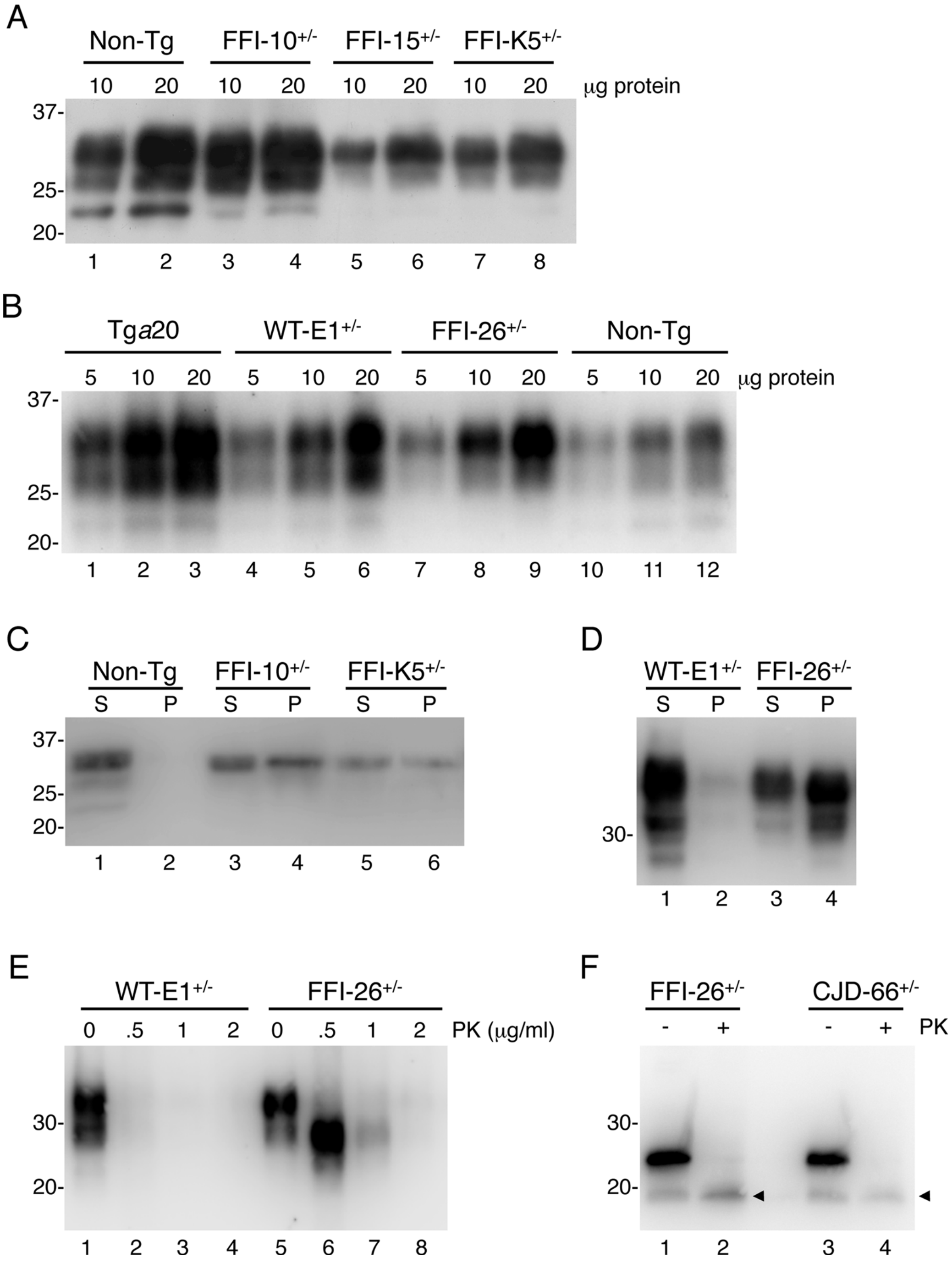

Fig 1. Mutant PrP in the brains of Tg(FFI) mice is insoluble and mildly protease-resistant. (A, B) The indicated amounts of total proteins extracted from the brains of a C57BL/6J mouse (non-Tg, 360 days old), a Tg(FFI-10$^{+/-}$)/*Prnp*$^{0/0}$ (87 days old), a Tg(FFI-15$^{+/-}$)/*Prnp*$^{0/0}$ (32 days old) and a Tg(FFI-K5$^{+/-}$)/*Prnp*$^{0/0}$ (601 days old) (panel A), and from a Tg*a*20 (113 days old), a Tg(WT-E1$^{+/-}$)/*Prnp*$^{0/0}$ (460 days old), a Tg(FFI-26$^{+/-}$)/*Prnp*$^{0/0}$ (227 days old) and a non-Tg mouse (307 days old) (panel B), were analyzed by Western blot with monoclonal antibody 12B2. (C, D) Brain lysates prepared from mice of the following genotypes and ages were ultracentrifuged at 186,000 x g for 40 min, and PrP in the supernatants (S lanes) and pellets (P lanes) was analyzed by Western blotting using the 12B2 antibody: non-Tg, 61 days; Tg(FFI-10$^{+/-}$)/*Prnp*$^{0/0}$, 58 days; Tg(FFI-K5$^{+/-}$)/*Prnp*$^{0/0}$, 287 days; Tg(WT-E1$^{+/-}$)/*Prnp*$^{0/0}$, 276 days; Tg(FFI-26$^{+/-}$)/*Prnp*$^{0/0}$, 371 days. (E) Brain lysates from the Tg(WT-E1$^{+/-}$)/*Prnp*$^{0/0}$ and Tg(FFI-26$^{+/-}$)/*Prnp*$^{0/0}$ mice used in D were incubated with 0–2 μg/ml of PK for 30 min at 37°C, and PrP was visualized by Western blotting using antibody 12B2. The undigested samples (0 μg/ml PK) represent 25 μg of protein, and the other samples 100 μg. (F) Brain lysates from the Tg(FFI-26$^{+/-}$)/*Prnp*$^{0/0}$ mouse and a Tg(CJD-66$^{+/-}$)/*Prnp*$^{0/0}$ mouse (322 days old) were incubated with 0 or 0.5 μg/ml of PK as in E, followed by incubation with PNGaseF and Western blot analysis with antibody 12B2. The arrowheads indicate the PK-resistant deglycosylated PrP bands. Molecular size markers are given in kDa.

doi:10.1371/journal.ppat.1004796.g001

		BOUTS (number)		
		REM Sleep	NREM Sleep	Wakefulness
Light (hours 1 - 12)	non-Tg/*Prnp*$^{+/+}$	43.38 ± 3.41	99.85 ± 5.90	62.50 ± 6.08
	non-Tg/*Prnp*$^{0/0}$	45.30 ± 3.25	110.90 ± 3.00	72.82 ± 5.39
	Tg(FFI)/*Prnp*$^{+/0}$	41.63 ± 3.37	127.54 ± 8.67	75.14 ± 9.22
	Tg(FFI)/*Prnp*$^{0/0}$	22.67 ± 3.81	119.46 ± 9.69	86.66 ± 9.39
Dark (hours 13 - 24)	non-Tg/*Prnp*$^{+/+}$	8.63 ± 2.79	72.75 ± 7.16	49.63 ± 6.48
	non-Tg/*Prnp*$^{0/0}$	14.80 ± 1.79	64.40 ± 5.62	41.05 ± 3.04
	Tg(FFI)/*Prnp*$^{+/0}$	9.50 ± 1.96	72.14 ± 10.11	48.24 ± 7.33
	Tg(FFI)/*Prnp*$^{0/0}$	15.67 ± 3.36	86.10 ± 7.83	56.76 ± 3.84

		BOUT DURATION (min)		
		REM Sleep	NREM Sleep	Wakefulness
Light (hours 1 - 12)	non-Tg/*Prnp*$^{+/+}$	1.29 ± 0.05	3.62 ± 0.20	6.46 ± 0.76
	non-Tg/*Prnp*$^{0/0}$	1.26 ± 0.05	3.38 ± 0.18	5.35 ± 0.61
	Tg(FFI)/*Prnp*$^{+/0}$	1.29 ± 0.07	2.93 ± 0.33	5.13 ± 0.55
	Tg(FFI)/*Prnp*$^{0/0}$	1.03 ± 0.12	2.76 ± 0.28	5.56 ± 0.84
Dark (hours 13 - 24)	non-Tg/*Prnp*$^{+/+}$	0.58 ± 0.12	2.55 ± 0.28	15.88 ± 1.85
	non-Tg/*Prnp*$^{0/0}$	0.80 ± 0.05	2.84 ± 0.26	18.67 ± 1.81
	Tg(FFI)/*Prnp*$^{+/0}$	0.51 ± 0.09	2.31 ± 0.24	17.93 ± 2.23
	Tg(FFI)/*Prnp*$^{0/0}$	0.61 ± 0.09	2.77 ± 0.34	9.89 ± 0.93

		TRANSITIONS (number/h)
Light (hours 1 - 12)	non-Tg/*Prnp*$^{+/+}$	27.33 ± 1.30
	non-Tg/*Prnp*$^{0/0}$	30.88 ± 1.54
	Tg(FFI)/*Prnp*$^{+/0}$	37.40 ± 2.57
	Tg(FFI)/*Prnp*$^{0/0}$	45.70 ± 3.43
Dark (hours 13 - 24)	non-Tg/*Prnp*$^{+/+}$	21.00 ± 2.24
	non-Tg/*Prnp*$^{0/0}$	18.88 ± 1.68
	Tg(FFI)/*Prnp*$^{+/0}$	24.03 ± 3.47
	Tg(FFI)/*Prnp*$^{0/0}$	34.82 ± 4.23

Fig 2. Sleep architecture. Values are the mean ± SEM of 8 non-Tg/*Prnp*$^{+/+}$, 10 non-Tg/*Prnp*$^{0/0}$ mice, 8 Tg(FFI-26)/*Prnp*$^{+/0}$ mice and 9 Tg(FFI-26)/*Prnp*$^{0/0}$ mice. The grey areas indicate the dark portion of the light-dark cycle. *p ≤ 0.05; **p ≤ 0.01 (mixed model for repeated measures followed by between-strain one-way ANOVA with Bonferroni's correction).

doi:10.1371/journal.ppat.1004796.g002

In 8 out of 9 Tg(FFI)/*Prnp*$^{0/0}$ mice, entry into REM sleep was abnormal. Tg(FFI)/*Prnp*$^{0/0}$ mice entered REM sleep directly from wakefulness in 24.6 ± 6.5% of REM epochs, instead of going through NREM sleep, as normally occurs (Fig 3). This was never observed in the other groups of mice.

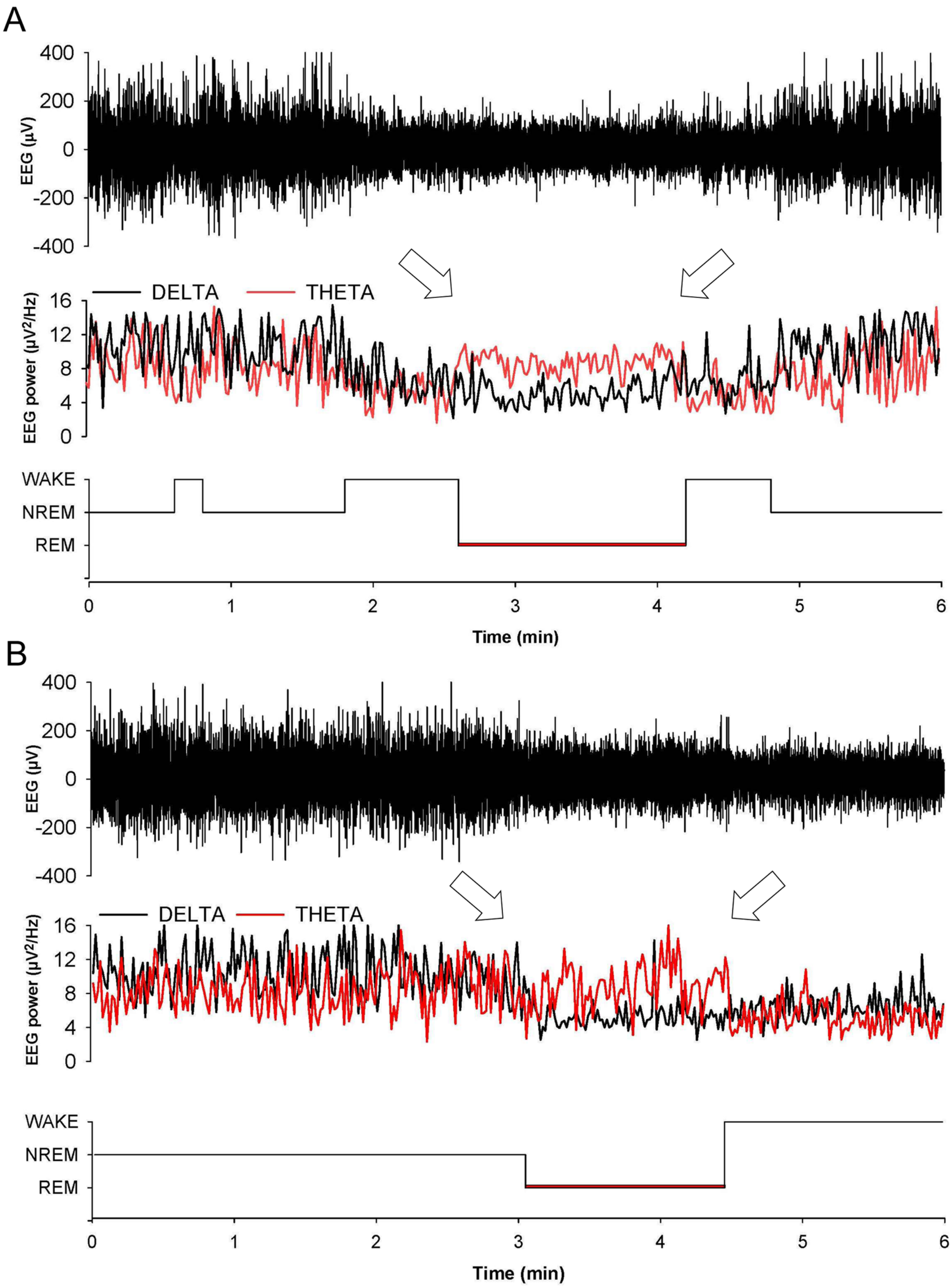

Fig 3. Entry in REM sleep is abnormal in Tg(FFI) mice. Whereas a Tg(FFI-26)/*Prnp*$^{0/0}$ mouse (A) enters REM sleep directly from wakefulness (left arrow), as shown by the hypnogram (lower trace), in a non-Tg/*Prnp*$^{+/+}$ mouse REM sleep is preceded by an episode of NREM sleep (B). Top to bottom: EEG (electroencephalogram), EEG power in the delta (0.5–4 Hz, black line) and theta (6–9 Hz, red line) bands, and the related hypnogram. Arrows indicate the beginning and end of a REM sleep phase.

doi:10.1371/journal.ppat.1004796.g003

Slow-wave activity (SWA) during NREM sleep (a measure of sleep drive and depth [17]) and EEG spindles (which characterize this sleep phase) were reduced in Tg(FFI)/*Prnp*$^{0/0}$ mice. Although the amount of NREM sleep was not reduced in Tg(FFI)/*Prnp*$^{0/0}$ mice in comparison to the other mice, NREM sleep SWA was significantly less in Tg(FFI)/*Prnp*$^{0/0}$ mice than in both non-Tg/*Prnp*$^{+/+}$ and non-Tg/*Prnp*$^{0/0}$ mice during the first portion of the light phase (Fig 4). The density of EEG spindles during NREM sleep in the light phase was lower in Tg(FFI)/*Prnp*$^{0/0}$ mice (38.4 ± 7.5 spindles/h) than in both non-Tg/*Prnp*$^{+/+}$ and non-Tg/*Prnp*$^{0/0}$ mice (184.4 ± 11.0 and 148.0 ± 9.1 spindles/h, respectively; $p < 0.01$ by one-way ANOVA $F_{3,31} = 35.587$). The density of spindles in Tg(FFI)/*Prnp*$^{+/0}$ mice (106.8 ± 14.3 spindles/h) was intermediate between that of non-Tg/*Prnp*$^{+/+}$ and Tg(FFI)/*Prnp*$^{0/0}$ mice, and significantly different from both.

Besides starting abnormally, in Tg(FFI)/*Prnp*$^{0/0}$ mice REM sleep differed in amount (Fig 4), and its EEG power in the theta (6–9 Hz) band (the EEG hallmark of rodent REM sleep) was significantly lower than in the other groups. The loss of REM sleep in Tg(FFI)/*Prnp*$^{0/0}$ mice was due to a reduction in the number of REM sleep bouts, but not their duration (Fig 2). During the light phase, REM sleep theta power was 11.8 ± 0.4% (of REM sleep EEG total power) in Tg(FFI)/*Prnp*$^{0/0}$ mice, 13.7 ± 0.4% in non-Tg/*Prnp*$^{+/+}$, and 13.2 ± 0.3% in non-Tg/*Prnp*$^{0/0}$ mice (mean ± SEM; $p < 0.05$ and $p < 0.001$, Tg(FFI)/*Prnp*$^{0/0}$ vs. non-Tg/*Prnp*$^{+/+}$ and non-Tg/*Prnp*$^{0/0}$, respectively; one-way ANOVA with Bonferroni's correction). During the dark phase, REM sleep theta power was significantly lower in both Tg(FFI)/*Prnp*$^{0/0}$ and Tg(FFI)/*Prnp*$^{+/0}$ than in non-Tg/*Prnp*$^{+/+}$ mice (11.8 ± 0.5%, 11.9 ± 0.5% and 13.9 ± 0.5% respectively; $p < 0.05$).

Since additional alterations may become apparent when the sleep drive is increased, we investigated the response to sleep deprivation. Mice were kept awake during the first 6 h of the light phase by gentle handling, then allowed to sleep freely in the following 18 h. REM and NREM sleep, shown in Fig 5, was calculated hourly for each animal as the difference between the time spent in REM or NREM sleep during and after sleep deprivation, and the amount spent in the corresponding hour during baseline conditions (undisturbed). The hour-by-hour differences were then summed to give a cumulative curve.

Nontransgenic and Tg(FFI)/*Prnp*$^{+/0}$ mice lost the same amount of REM sleep during deprivation (Fig 5A). By the end of the recording period (i.e. 18 h after the end of the sleep deprivation period), non-Tg/*Prnp*$^{+/+}$ and non-Tg/*Prnp*$^{0/0}$ mice fully recovered the REM sleep lost, whereas Tg(FFI)/*Prnp*$^{+/0}$ mice did not (Figs 4 and 5A). During sleep deprivation, Tg(FFI)/*Prnp*$^{0/0}$ mice lost less REM sleep than all other mouse lines, because REM sleep was already markedly reduced in these mice in basal conditions (Fig 4). In the next 18 h, Tg(FFI)/*Prnp*$^{0/0}$ mice slept as much as in undisturbed conditions, having little loss of REM sleep to recover (Figs 4 and 5A).

Mice of all genotypes lost the same amount of NREM sleep during sleep deprivation. During the next 18 h, they did not fully recover (Fig 5B) and compensated the loss of NREM sleep with an increase in the power of the EEG delta band in the first 6 h of recovery (Fig 4), as previously shown for rodents deprived of sleep by gentle handling for a short time [18].

EEG activity was altered in Tg(FFI)/*Prnp*$^{0/0}$ and Tg(FFI)/*Prnp*$^{+/0}$ mice. This consisted of bursts of high-voltage polyphasic complexes, similar to those described in Tg(CJD) mice expressing D177N/V128 PrP [14], with frequency peaking at about 7 Hz. This activity was almost equally distributed during the light and dark parts of the light/dark cycle. In Tg(FFI)/*Prnp*$^{0/0}$ mice polyphasic complexes were present in respectively 8.8 ± 3.4% and 12.0 ± 3.7% of the epochs of the light and dark parts of the light-dark cycle. In Tg(FFI)/*Prnp*$^{+/0}$ polyphasic complexes were present in 0.60 ± 0.24% and 1.15 ± 0.60% of epochs of the light and dark phases. Polyphasic complexes were present in 9.6 ± 3.1%, 2.1 ± 1.0% and 16.4 ± 5.2% of epochs scored respectively as REM sleep, NREM sleep and wakefulness, in Tg(FFI)/*Prnp*$^{0/0}$ mice. These percentages were 0.4 ± 0.2%, 0.5 ± 0.3% and 1.1 ± 0.5% in Tg(FFI)/*Prnp*$^{+/0}$ mice. No pathological activity was detected in non-Tg mice.

		Baseline		Sleep Deprivation and Recovery	
**REM Sleep (% recording time)**					
**0-6 hours**	non-Tg/*Prnp*+/+	7.81 ± 0.77		0.00 ± 0.00	°°
	non-Tg/*Prnp*0/0	7.93 ± 0.59	*	0.00 ± 0.00	°°
	Tg(FFI)/*Prnp*+/0	7.53 ± 0.82	**	0.00 ± 0.00	°°
	Tg(FFI)/*Prnp*0/0	4.42 ± 0.79	*	0.00 ± 0.00	°°
**7-12 hours**	non-Tg/*Prnp*+/+	8.51 ± 0.71		10.72 ± 0.72	°
	non-Tg/*Prnp*0/0	8.86 ± 0.68		10.89 ± 0.56	°°
	Tg(FFI)/*Prnp*+/0	7.99 ± 0.75		9.71 ± 1.44	
	Tg(FFI)/*Prnp*0/0	3.92 ± 0.71	**	4.75 ± 0.65	**
**13-18 hours**	non-Tg/*Prnp*+/+	1.60 ± 0.44		4.25 ± 0.63	°
	non-Tg/*Prnp*0/0	2.72 ± 0.33		5.61 ± 0.84	°°
	Tg(FFI)/*Prnp*+/0	1.67 ± 0.42		2.25 ± 0.35	
	Tg(FFI)/*Prnp*0/0	2.28 ± 0.54		3.26 ± 0.65	
**19-24 hours**	non-Tg/*Prnp*+/+	1.46 ± 0.53		4.42 ± 0.55	°°
	non-Tg/*Prnp*0/0	2.54 ± 0.39		3.74 ± 0.56	
	Tg(FFI)/*Prnp*+/0	1.29 ± 0.29		2.95 ± 0.53	°
	Tg(FFI)/*Prnp*0/0	2.65 ± 0.55		2.88 ± 0.58	
**Wakefulness (% recording time)**					
**0-6 hours**	non-Tg/*Prnp*+/+	44.88 ± 3.13		91.30 ± 1.01	°°
	non-Tg/*Prnp*0/0	40.53 ± 1.78		93.81 ± 0.69	°°
	Tg(FFI)/*Prnp*+/0	46.42 ± 3.19		90.99 ± 1.07	°°
	Tg(FFI)/*Prnp*0/0	47.67 ± 2.85		91.42 ± 1.72	°°
**7-12 hours**	non-Tg/*Prnp*+/+	38.20 ± 2.50		32.71 ± 1.14	
	non-Tg/*Prnp*0/0	40.65 ± 1.75	**	29.89 ± 1.36	°°
	Tg(FFI)/*Prnp*+/0	39.59 ± 2.59	*	33.94 ± 3.03	°
	Tg(FFI)/*Prnp*0/0	51.30 ± 2.95	*	39.40 ± 4.43	°
**13-18 hours**	non-Tg/*Prnp*+/+	72.10 ± 2.65		61.25 ± 4.03	°
	non-Tg/*Prnp*0/0	68.58 ± 3.02		58.35 ± 3.20	* °°
	Tg(FFI)/*Prnp*+/0	76.01 ± 2.29		72.15 ± 1.70	**
	Tg(FFI)/*Prnp*0/0	65.23 ± 3.53		54.51 ± 4.07	°
**19-24 hours**	non-Tg/*Prnp*+/+	68.96 ± 3.38		59.78 ± 3.95	
	non-Tg/*Prnp*0/0	69.48 ± 2.06		66.78 ± 1.94	
	Tg(FFI)/*Prnp*+/0	72.15 ± 2.95		67.87 ± 3.84	
	Tg(FFI)/*Prnp*0/0	62.03 ± 3.04		59.06 ± 3.38	

		Baseline		Sleep Deprivation and Recovery	
**NREM Sleep (% recording time)**					
**0-6 hours**	non-Tg/*Prnp*+/+	47.32 ± 2.73		8.70 ± 1.01	°°
	non-Tg/*Prnp*0/0	51.53 ± 1.47		6.19 ± 0.69	°°
	Tg(FFI)/*Prnp*+/0	46.05 ± 2.49		8.99 ± 1.04	°°
	Tg(FFI)/*Prnp*0/0	47.43 ± 2.50		8.09 ± 1.75	°°
**7-12 hours**	non-Tg/*Prnp*+/+	53.28 ± 2.24		56.58 ± 1.04	
	non-Tg/*Prnp*0/0	50.49 ± 1.86		59.22 ± 1.56	°°
	Tg(FFI)/*Prnp*+/0	52.42 ± 2.05		56.35 ± 1.73	°
	Tg(FFI)/*Prnp*0/0	44.35 ± 2.94		55.80 ± 4.07	°
**13-18 hours**	non-Tg/*Prnp*+/+	26.31 ± 2.35		34.50 ± 3.55	
	non-Tg/*Prnp*0/0	28.70 ± 2.74		36.01 ± 2.62	°°
	Tg(FFI)/*Prnp*+/0	22.30 ± 2.04		25.51 ± 1.58	**
	Tg(FFI)/*Prnp*0/0	31.94 ± 3.03		41.84 ± 3.82	°
**19-24 hours**	non-Tg/*Prnp*+/+	29.58 ± 3.23		35.80 ± 3.49	
	non-Tg/*Prnp*0/0	27.98 ± 1.79		29.48 ± 1.68	
	Tg(FFI)/*Prnp*+/0	26.54 ± 2.81		29.15 ± 3.45	
	Tg(FFI)/*Prnp*0/0	35.00 ± 2.77		37.68 ± 3.00	
**Slow Wave Activity during NREM Sleep (% from 24 h baseline)**					
**0-6 hours**	non-Tg/*Prnp*+/+	13.14 ± 1.91		20.16 ± 4.35	
	non-Tg/*Prnp*0/0	12.57 ± 1.66	** * *	27.90 ± 6.71	°
	Tg(FFI)/*Prnp*+/0	3.92 ± 3.49	**	23.30 ± 3.86	°
	Tg(FFI)/*Prnp*0/0	-1.16 ± 1.24		25.91 ± 5.40	°°
**7-12 hours**	non-Tg/*Prnp*+/+	-2.91 ± 1.03		8.89 ± 3.66	°
	non-Tg/*Prnp*0/0	-5.72 ± 1.13		9.19 ± 1.69	°°
	Tg(FFI)/*Prnp*+/0	-5.60 ± 1.38		8.77 ± 1.83	°°
	Tg(FFI)/*Prnp*0/0	-6.51 ± 1.78		6.50 ± 2.74	°°
**13-18 hours**	non-Tg/*Prnp*+/+	-2.06 ± 2.13		-2.10 ± 3.30	
	non-Tg/*Prnp*0/0	5.29 ± 2.43		-2.49 ± 2.07	°
	Tg(FFI)/*Prnp*+/0	1.93 ± 3.23		4.22 ± 2.87	
	Tg(FFI)/*Prnp*0/0	5.48 ± 2.29		2.39 ± 2.45	
**19-24 hours**	non-Tg/*Prnp*+/+	4.70 ± 1.85		-0.96 ± 3.59	
	non-Tg/*Prnp*0/0	6.06 ± 1.95		-0.69 ± 2.75	°
	Tg(FFI)/*Prnp*+/0	9.16 ± 3.45		8.95 ± 3.52	
	Tg(FFI)/*Prnp*0/0	3.93 ± 1.82		3.80 ± 3.82	

Fig 4. Amount of sleep and EEG delta power during NREM sleep. Values are the mean ± SEM of 8 non-Tg/*Prnp*$^{+/+}$, 10 non-Tg/*Prnp*$^{0/0}$, 8 Tg(FFI-26)/*Prnp*$^{+/0}$ and 9 Tg(FFI-26)/*Prnp*$^{0/0}$ mice. The grey areas indicate the dark portion of the light-dark cycle. *,°$p \leq 0.05$; **,°°$p \leq 0.01$. A mixed model for repeated measures was used. Between-strain comparisons (*) were done by one-way ANOVA with Bonferroni's correction. Within-condition comparisons (°) were done by paired Student's t test.

doi:10.1371/journal.ppat.1004796.g004

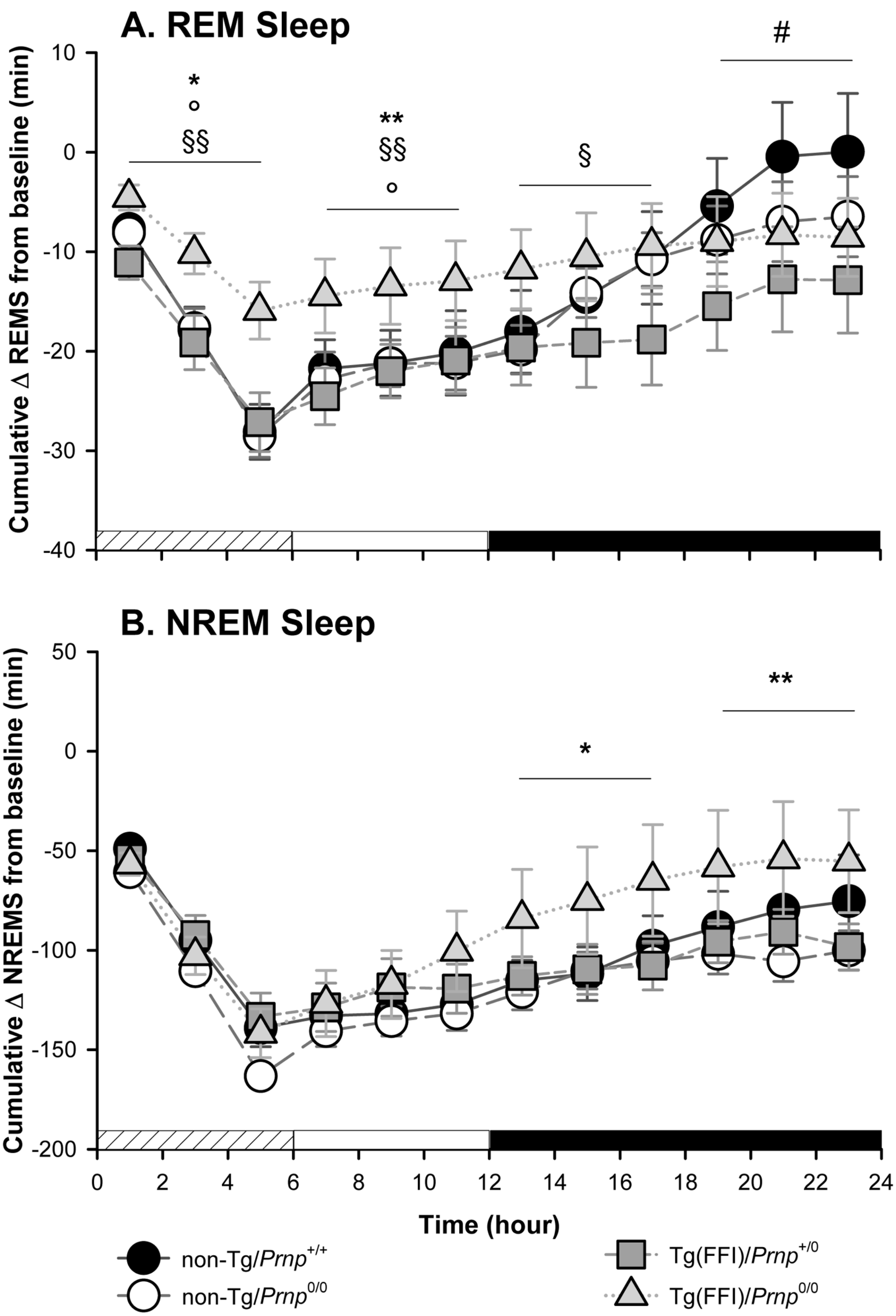

Fig 5. Tg(FFI) mice show an altered response to sleep deprivation. Time course of the loss and recovery of time spent in rapid eye movement (REM) (A) and non-rapid eye movement (NREM) (B) sleep, during and after sleep deprivation. Values were from 8 non-Tg/*Prnp*$^{+/+}$, 10 non-Tg/*Prnp*$^{0/0}$, 9 Tg(FFI-26)/*Prnp*$^{0/0}$ and 8 Tg(FFI-26)/*Prnp*$^{+/0}$. Mice were kept awake during the first 6 h of the light phase (crosshatched bar) by gentle handling, and allowed to sleep freely in the next 18 h. The black bar indicates the dark portion of the light-dark cycle. REM and NREM sleep were calculated hourly for each animal as the difference between the amount of time spent in a given state (REM or NREM sleep) during and after sleep deprivation, and the amount spent in the

corresponding hour during baseline conditions (undisturbed). The hour-by-hour differences were then summed to obtain a cumulative curve. Data (means ± SEM) are presented in 2-h intervals. Single symbols: $p < 0.05$; double symbols: $p < 0.01$. *, Tg(FFI-26)/*Prnp*$^{0/0}$ vs non-Tg/*Prnp*$^{0/0}$; °, Tg(FFI-26)/*Prnp*$^{0/0}$ vs. non-Tg/*Prnp*$^{+/+}$; §, Tg(FFI-26)/*Prnp*$^{0/0}$ vs. Tg(FFI-26)/*Prnp*$^{+/0}$; #, Tg(FFI-26)/*Prnp*$^{+/0}$ vs. non-Tg/*Prnp*$^{+/+}$. A mixed model analysis of variance for repeated measures was done on 6 h blocks. Between-strains post-hoc comparisons by one-way ANOVA with Bonferroni correction: (panel A) 0–6 h: $F_{3,101}$ = 4.98, p = 0.003; 7–12 h: $F_{3,101}$ = 5.25, p = 0.002; 13–18 h: $F_{3,101}$ = 2.88, p = 0.05; 19–24 h: $F_{3,101}$ = 3.30, p = 0.023. (panel B) 0–6 h: $F_{3,101}$ = 1.01, p = 0.391; 7–12 h: $F_{3,101}$ = 1.78, p = 0.156; 13–18 h: $F_{3,101}$ = 3.76, p = 0.013; 19–24 h: $F_{3,101}$ = 3.97, p = 0.010.

doi:10.1371/journal.ppat.1004796.g005

Tg(FFI) Mice Develop Motor Dysfunction and Alterations of Recognition and Spatial Working Memory

Tg(FFI) mice had progressive neurological disease. They developed ataxia, with abnormal flexed posture of the hind legs, kyphosis, and foot clasp reflex (S2 Fig). The phenotype was evident from 202 ± 2 days (mean ± SEM, n = 58) in Tg(FFI-26) mice. As the disease progressed the mice lost weight, and were killed when unable to feed themselves, at 436 ± 4 days (n = 87). Prospective observation of individual mice indicated an average duration of the illness of 262 ± 4 days (n = 32).

To check the earliest appearance of motor dysfunction, Tg(FFI-26) mice were tested on the accelerating Rotarod. They performed well until 90 days of age, indicating normal development of motor function. From 110 days on, however, the mutant mice showed a significantly shorter latency to fall than nontransgenic littermates; their performance worsened with aging until they became unable to stay on the rod (Fig 6A).

A similar but less aggressive neurological illness was seen in Tg(FFI-10) mice expressing mutant PrP at lower levels. Neurological signs were evaluated at a single time in a cohort of hemi- and homozygous Tg(FFI-10) littermates of different ages. There was no disease in hemizygous Tg(FFI-10) mice younger than 328 days, but 23 out of 37 (62%) mice between 328 and 757 days showed mild neurological disease. Prospective observation indicated that these mice never reached a debilitating stage and survived as long as non-Tg littermates. In addition, some hemizygous Tg(FFI-10) animals remained free of neurological signs, suggesting incomplete penetrance when the mutant PrP was expressed at wild-type levels.

In contrast, all homozygous Tg(FFI-10) mice older than 290 days had neurological disease, and reached a terminal stage at 698 ± 26 days (n = 14), indicating a profound effect of transgene zygosity on the manifestation and time course of the illness. Confirming this, the Rotarod task showed age-dependent, transgene-dose-related motor dysfunction in Tg(FFI-10) mice (Fig 6B). The non-breeding Tg(FFI-28) founder expressing PrP at ~5X died with neurological symptoms at 477 days, whereas Tg(FFI) lines with mutant PrP levels below 1X (Table 1) never developed neurological disease. Thus the appearance of neurological illness and its rate of progression were correlated with the expression level of mutant PrP, similar to other mouse models of genetic prion disease [13, 14, 19–21].

Co-expression of WT PrP had no effect on motor dysfunction, in contrast to the mitigating effect on the sleep abnormalities. For example, in a group of littermates consisting of 9 Tg(FFI-26)/*Prnp*$^{0/0}$, 10 Tg(FFI-26)/*Prnp*$^{+/0}$ and 8 Tg(FFI-26)/*Prnp*$^{+/+}$ mice, symptom onset was at 207 ± 13, 216 ± 12 and 220 ± 11 days respectively ($F_{2,24}$ = 0.29; p = 0.75 by one-way ANOVA). There were also no differences in Rotarod performance (Fig 6C).

We found alterations in long-term recognition and spatial working memory too in Tg(FFI) mice, tested in the novel object recognition task and eight-arm radial maze. To avoid confounding effects due to the motor deficit that develops in older mice, we tested Tg(FFI-26) animals younger than 100 days. Mice were impaired in long-term memory, as shown by the lower discrimination index in the object recognition task compared to non-Tg mice (Fig 7A). They also performed poorly in the eight-arm radial maze, which tests spatial working memory,

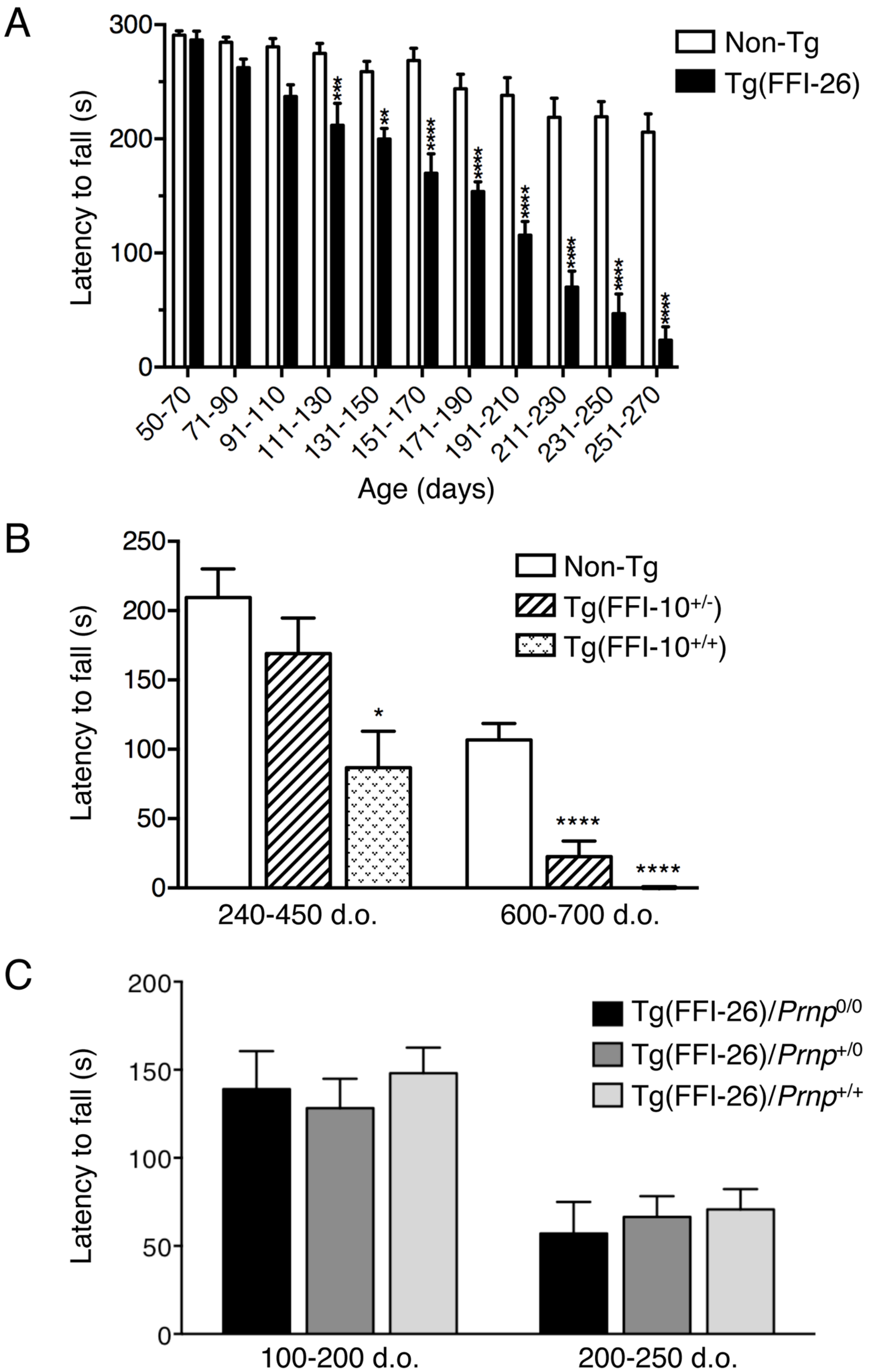

Fig 6. Tg(FFI) mice develop motor dysfunction which is not rescued by co-expression of wild-type PrP. (A) Groups of 7–12 Tg(FFI-26$^{+/-}$)/*Prnp*$^{0/0}$ and 9–13 non-Tg/*Prnp*$^{0/0}$ littermates were tested on a Rotarod at the ages indicated. Each mouse was tested three times, and the mean latency to fall was calculated. Bars indicate the mean ± SEM of latency to fall (s); $F_{10,192} = 10.82$, $p < 0.0001$ by two-way ANOVA; **$p < 0.01$ and ****$p < 0.0001$ Šidàk's post hoc test. (B) Groups of 6–19 (240–450 days old) and 10–11 (600–700 days old)

Tg(FFI-10$^{+/-}$)/*Prnp*$^{0/0}$, Tg(FFI-10$^{+/+}$)/*Prnp*$^{0/0}$ and non-Tg/*Prnp*$^{0/0}$ littermates were tested on a Rotarod. Bars indicate the mean ± SEM of latency to fall (s); $F_{2,28} = 34.05$, $p < 0.0001$ by one-way ANOVA; *$p < 0.05$ and ****$p < 0.0001$ vs. non-Tg; Tukey's post hoc test. (C) Groups of 9 Tg(FFI-26$^{+/-}$)/*Prnp*$^{0/0}$, 10 Tg(FFI-26$^{+/-}$)/*Prnp*$^{+/0}$, and 8 Tg(FFI-26$^{+/-}$)/*Prnp*$^{+/+}$ littermates were tested on a Rotarod and the ages indicated. $F_{2,45} = 0.3374$; $p = 0.7154$ by two-way ANOVA.

doi:10.1371/journal.ppat.1004796.g006

making significantly more errors in the first eight training trials than controls (Fig 7B). Latency to complete the test was longer in Tg(FFI) than non-Tg mice (Fig 7C). This may reflect an impairment in choice-making, since there were no significant differences in the number of total movements in the open field (total line crossings: non-Tg = 304 ± 13; Tg(FFI-26) = 283 ± 23; mean ± SEM), confirming no motor deficit at this stage.

Tg(FFI) Mice Have Thalamic and Cerebellar Atrophy, PrP Deposits and Gliosis

We used magnetic resonance imaging (MRI) to investigate the effects of the FFI mutation on brain structure. There were no significant differences between the whole-brain volumes of Tg (FFI-26) and non-Tg littermates at 80 days of age (non-Tg = 464 ± 6 mm^3; Tg(FFI-26) = 451 ± 5 mm^3; mean ± SEM, n = 5–6; $p = 0.0823$ by Mann-Whitney test). In Tg(FFI-26) mice older than 400 days, the whole-brain volume was 12% smaller than controls (non-Tg = 493 ± 4 mm^3; Tg(FFI-26) = 436 ± 6 mm^3; mean ± SEM, n = 9; $p < 0.0001$ by Mann-Whitney test). Analysis of individual brain areas showed that the thalamic and cerebellar volumes were significantly smaller in Tg(FFI-26) mice than in non-Tg littermates, but there were no differences in the other brain regions (Fig 8).

Neuropathological examination of Tg(FFI) mice showed PrP deposition in the form of diffuse "synaptic-type" immunoreactivity in several regions. These deposits were most prominent in the entorhinal and pyriform cortex, cingulate gyrus, hippocampal formation, thalamus, caudatum, putamen, amygdala and the molecular layer of the cerebellar cortex (Fig 9). Synaptic-type deposits were also present in the other cortical areas and the septum. Dot-like and small round PrP-immunoreactive profiles were seen in several subcortical structures, including the stria terminalis, fimbria and thalamus (Fig 9). Strongly immunoreactive fiber tracts were observed in the stria terminalis and in the stratum lucidum of CA3, corresponding to the hippocampal mossy fibers. Diffuse intraneuronal PrP immunoreactivity was present in the mesencephalic trigeminal nucleus, the vestibular nucleus and the lateral dorsal nucleus of the thalamus. The neurons of the mesencephalic trigeminal nucleus were outlined by dot-like immunoreactive profiles that were also scattered in the neuropil (Fig 9J). The PrP deposits were not fluorescent after thioflavin S staining, indicating that they did not contain amyloid fibrils. No spongiform-like changes were seen. Immunohistochemistry with the anti-GFAP antibody revealed astrogliosis mainly in the hippocampus, external layer of the cerebral cortex, and cerebellum (S3A-S3F Fig). Staining with the anti-Iba1 antibody showed microgliosis mainly in the hippocampus, cerebral cortex and periaqueductal gray (S3G-S3L Fig).

Tg(FFI) Neurons Show Morphological Abnormalities of the Golgi with Accumulation of Mutant PrP

Electron microscopy (EM) of Tg(FFI) brains detected neuronal ultrastructure abnormalities in several regions, including the neocortex, hippocampus, thalamus and cerebellum. These included autophagosomes, autophagolysosomes and increased amounts of lipofuscin (Fig 10A–10D). The most marked finding, however, was alteration of the Golgi complex, whose cisternae appeared swollen and 'swirled', often forming an onion-like structure (Fig 10E–10G). Three-

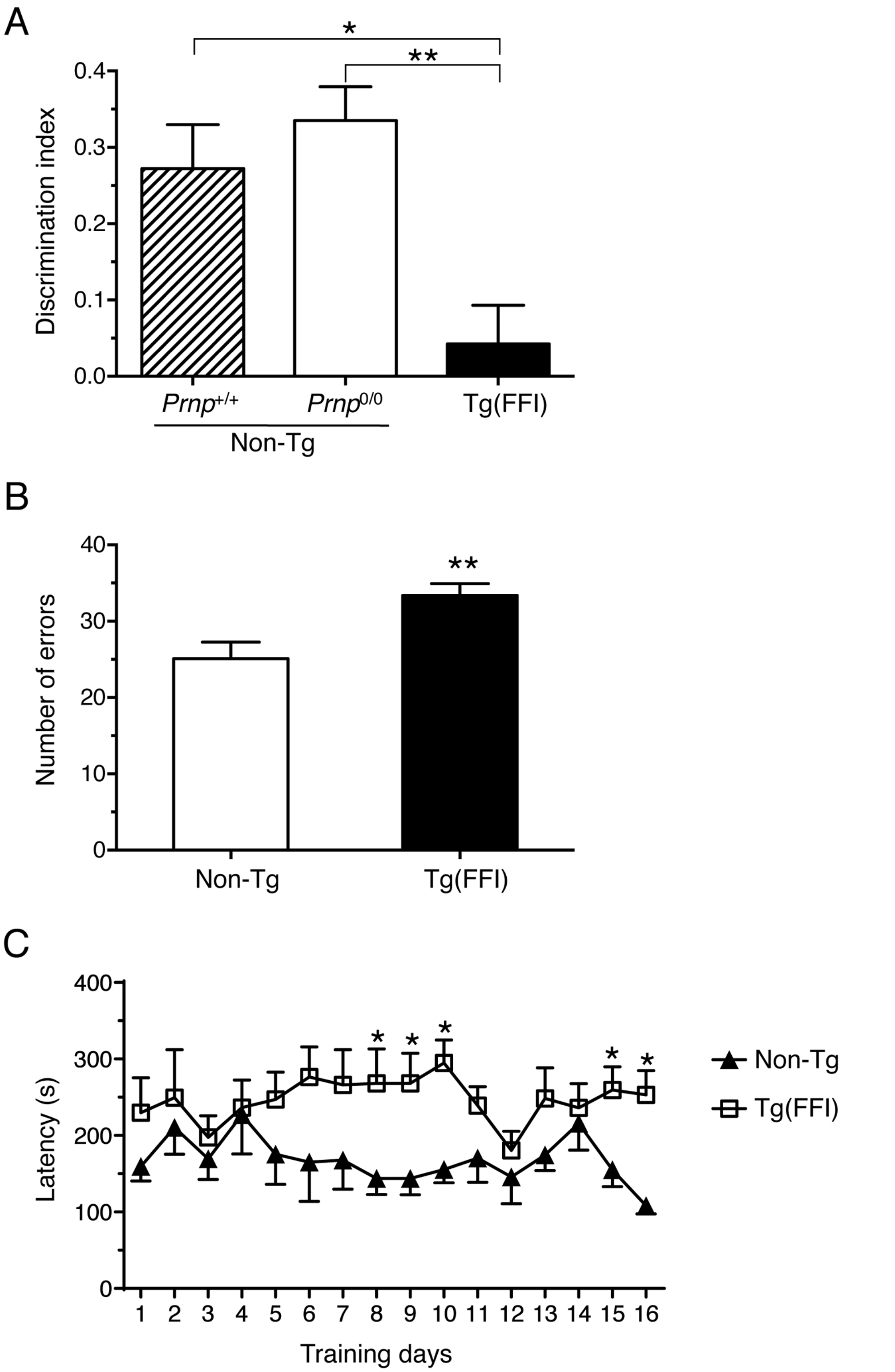

Fig 7. Tg(FFI) mice show recognition and spatial working memory impairment. (A) Performance in the novel object recognition task was expressed as a discrimination index (see Experimental Procedures). Histograms indicate the mean ± SEM of 10 non-Tg/*Prnp*$^{+/+}$, 10 non-Tg/*Prnp*$^{0/0}$, and 8 Tg(FFI-26$^{+/-}$)/*Prnp*$^{0/0}$ aged 70 days; $F_{2,25} = 8.3$ $p = 0.017$ by one-way ANOVA; *$p < 0.05$, **$p < 0.01$, Tukey's post hoc test. (B) Histograms represent the mean ± SEM of total errors in the eight-arm radial maze in the first eight trials during

16 days of training, by the same non-Tg/*Prnp*$^{0/0}$ and Tg(FFI-26$^{+/-}$)/*Prnp*$^{0/0}$ mice used in A. $t_{16} = 3.0$; $p = 0.009$; $^{**}p < 0.01$ by Student's t test. (C) Values are the mean latency (± SEM) to complete the radial maze. $F_{15,240} = 19$; $p = 0.03$ by one-way ANOVA for repeated measures. $^{*}p < 0.05$ by Student's t test.

doi:10.1371/journal.ppat.1004796.g007

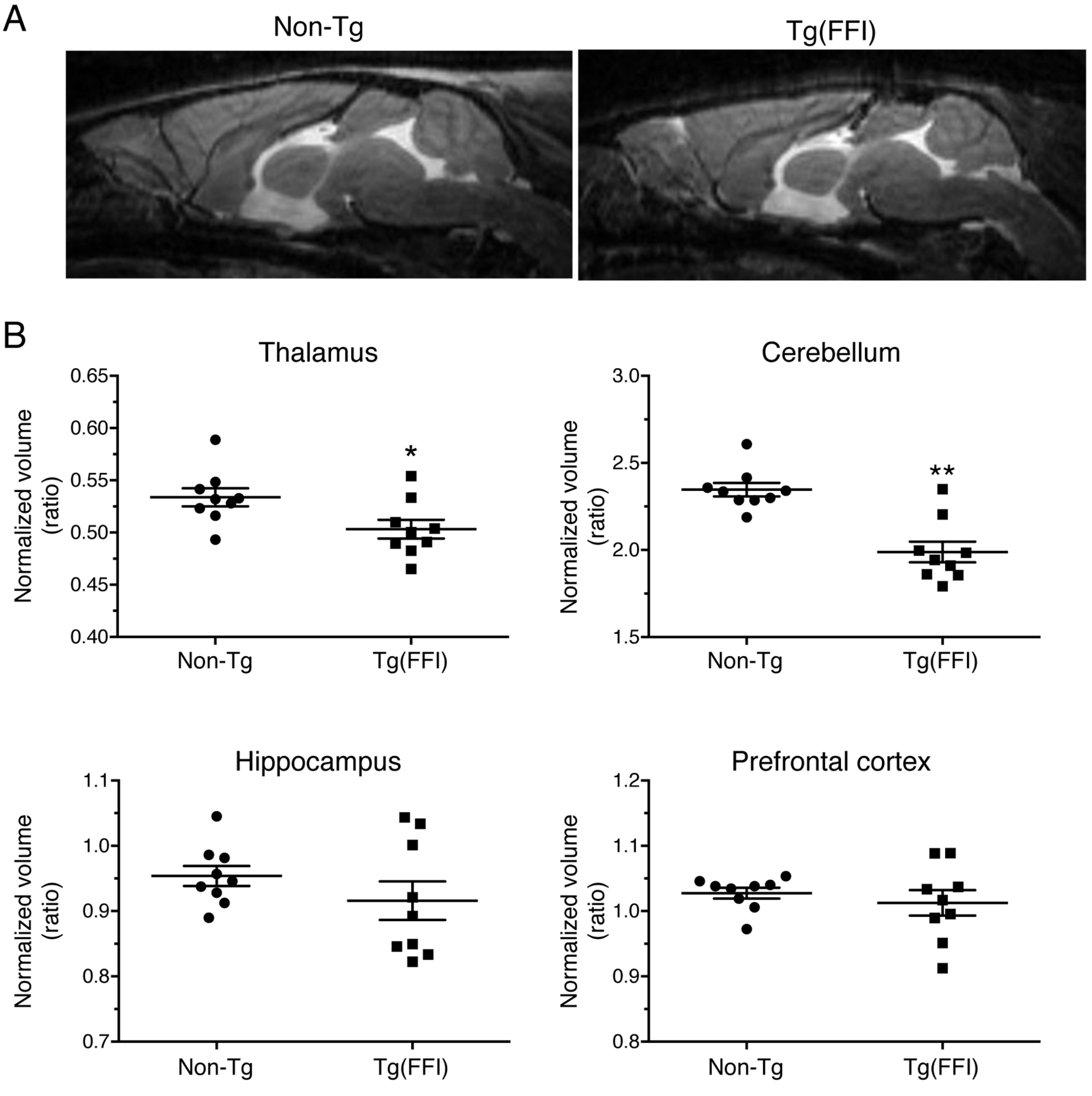

Fig 8. Tg(FFI) mice show thalamic and cerebellar atrophy. (A) Brain anatomy of a non-Tg/*Prnp*$^{0/0}$ and a Tg(FFI-26$^{+/-}$)/*Prnp*$^{0/0}$ mouse aged 460 days. Representative T2-weighted images (TE/TR = 50/2500 ms). (B) Volumes of individual brains areas of 9 Tg(FFI-26$^{+/-}$)/*Prnp*$^{0/0}$ and 9 non-Tg/*Prnp*$^{0/0}$ littermates aged between 408 and 498 days were quantified as described in the Experimental Procedures. To reduce interindividual variation, volumes were normalized on the values of the striatum which were the same in non-Tg and Tg(FFI) mice (non-Tg: 20.83 ± 0.30 mm^3, Tg(FFI): 20.09 ± 0.32 mm^3; mean ± SEM, $p = 0.22$ by Mann Whitney test). $^{*}p < 0.05$ and $^{**}p < 0.01$ vs. non-Tg by Mann Whitney test.

doi:10.1371/journal.ppat.1004796.g008

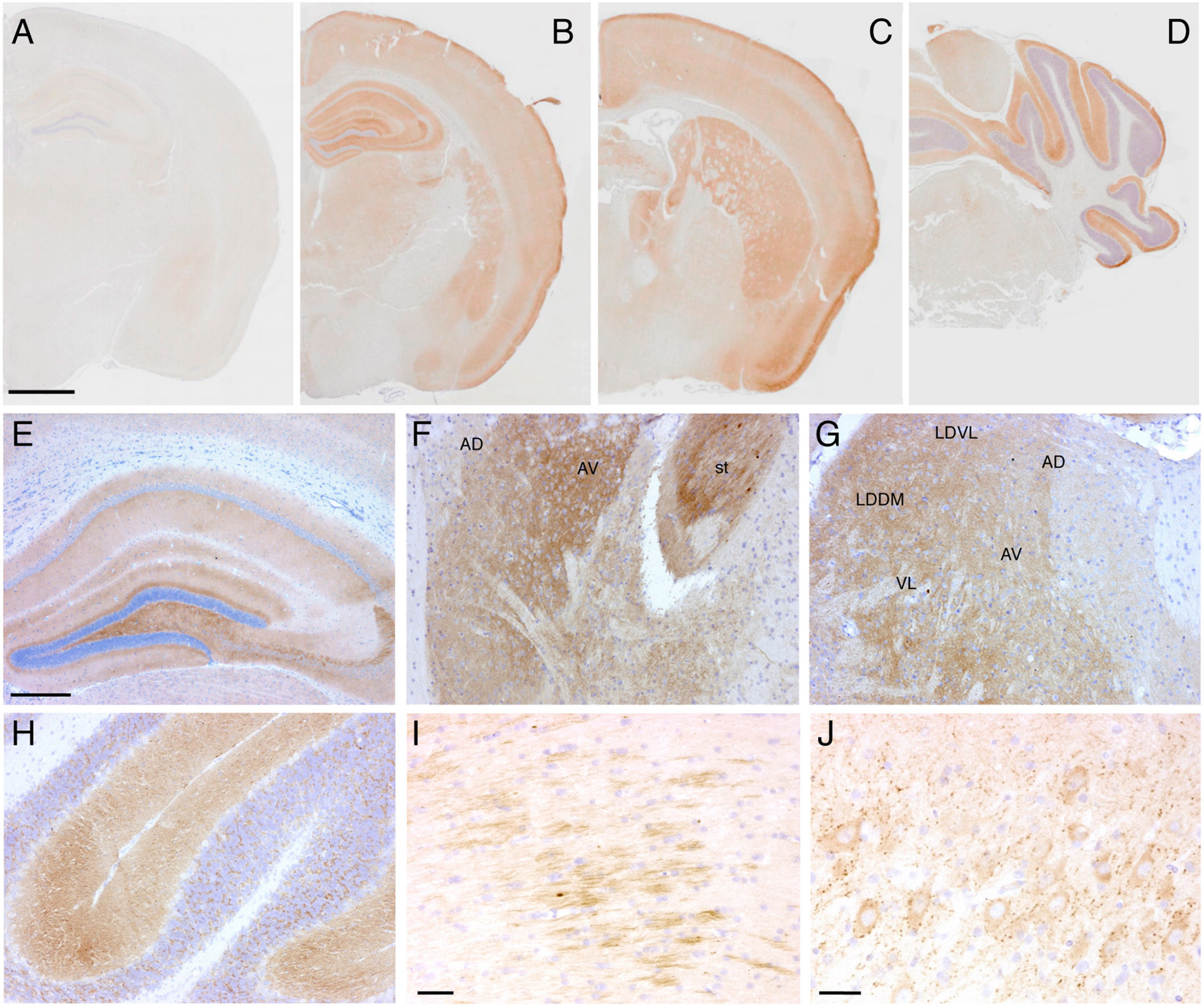

Fig 9. Tg(FFI) mice show cerebral accumulation of protease-resistant PrP. Immunohistochemical detection of PrP using monoclonal antibody 12B2 after PK digestion of sections in a 291-day-old Tg(WT-E1$^{+/-}$)/*Prnp*$^{0/0}$ mouse (A) and in three 338-day-old Tg(FFI-26$^{+/-}$)/*Prnp*$^{0/0}$ mice (B-J). The pattern of PrP deposition was either diffuse, as in the cerebral cortex, hippocampus, thalamus and molecular layer of the cerebellum (B-H), strip-like as in the fimbria (I), or dot-like as in the mesencephalic trigeminal nucleus (J). AD, anterodorsal thalamic nucleus; AV, anteroventral thalamic nucleus; st, stria terminalis; LDDM, laterodorsal thalamic nucleus, dorsomedial part; LDVL laterodorsal thalamic nucleus, ventrolateral part; VL, ventrolateral thalamic nucleus. Scale bars = 1 mm in A, B, C and D, 250 μm in E, F, G and H, and 125 μm in I and J. Results were similar using the 3F4 antibody in Tg(FFI-K5$^{+/-}$)/*Prnp*$^{0/0}$ mice expressing epitopically-tagged mutant PrP.

doi:10.1371/journal.ppat.1004796.g009

dimensional tomography confirmed the concentric isolated Golgi cisternae and showed invaginations of medial Golgi cisternae inside their lumen (Fig 10H and 10I). These abnormalities were never seen in Tg(WT-E1) and non-Tg controls.

To see whether the Golgi abnormalities in Tg(FFI) neurons were associated with intracellular accumulation of mutant PrP, we examined primary CGNs by immuno-gold EM using a published procedure [14]. The majority of WT PrP in granule neurons from non-Tg/*Prnp*$^{+/+}$ mice localized on the plasma membrane and in endosomes, with only a small fraction in the ER and Golgi (Fig 11A and 11D). In contrast, D177N/M128 PrP localized mostly in the Golgi of Tg(FFI) neurons (~75% vs. ~2.5% in control cells), with far fewer molecules on the plasma

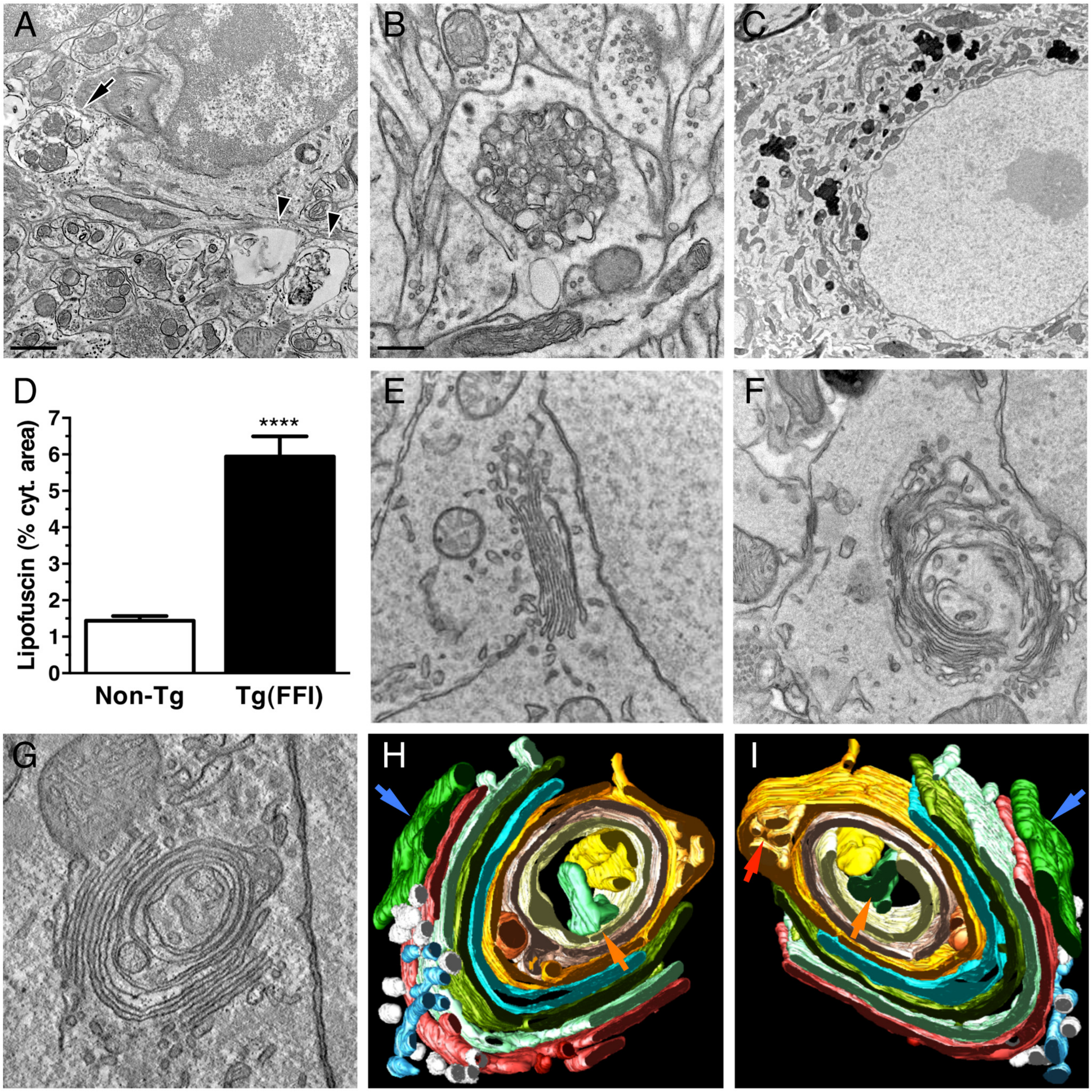

Fig 10. Ultrastructural abnormalities in Tg(FFI) neurons. (A) Autophagosomes in a hippocampal neuron (arrow) and the surrounding neuropil (arrowheads) of a Tg(FFI-$26^{+/-}$)/$Prnp^{0/0}$ mouse at 367 days. (B) Autophagosomes and autophagolysosomes in a dystrophic cerebellar neurite of a Tg(FFI-$26^{+/-}$)/$Prnp^{+/0}$ at 444 days. (C) Lipofuscin residual bodies in a thalamic neuron of a Tg(FFI-$26^{+/-}$)/$Prnp^{0/0}$ mouse at 367 days. (D) Quantification of lipofuscin residual bodies in the thalamus of three non-Tg and four Tg(FFI) mice aged between 292 and 444 days. Data are the mean ± SD. ****$p < 0.0001$ by Student's t test. Values from two non-Tg/$Prnp^{+/+}$ and one non-Tg/$Prnp^{0/0}$ mice, and from three Tg(FFI-$26^{+/-}$)/$Prnp^{0/0}$ and one Tg(FFI-$26^{+/-}$)/$Prnp^{+/0}$ mice were pooled. (E) Normal Golgi appearance in a hippocampal neuron of a non-Tg/$Prnp^{+/+}$ mouse at 280 days. Onion-like Golgi morphology in hippocampal (F) and thalamic (G) neurons of a Tg(FFI-$10^{+/-}$)/$Prnp^{0/0}$ mouse at 331 days. (H, I) Three-dimensional tomography reconstruction from virtual serial slices of the Golgi shown in G. The ER cisterna is colored green (blue arrows). The trans-most cisterna is in the center of the spherical Golgi (blue-green, orange arrows). Medial Golgi cisternae show invaginations of membranes inside their lumen (red arrow in panel I). The cis-most cisterna is not visible in this Golgi stack. Scale bars = 1 μm in A and C, and 0.5 μm in B and E, F and G.

doi:10.1371/journal.ppat.1004796.g010

membrane (~15% vs. ~85% in controls) (Fig 11B and 11D). The Golgi in these neurons were bigger than controls (Fig 11E). These abnormalities in PrP distribution and intracellular organelle morphology were strikingly different from those of Tg(CJD) neurons, in which we found dramatic swelling of the ER cisternae, with ER retention of mutant PrP (Fig 11C–11E) [14].

The Brains of Spontaneously Ill Tg(FFI) and Tg(CJD) Mice Do Not Contain Detectable Prion Infectivity

Intracerebral inoculation of brain homogenates from FFI and CJD^{178} patients induced prion disease in experimental animals [22–24], consistent with the contention that D178N PrP can spontaneously acquire an infectious structure. To test whether prion infectivity was generated *de novo* in the brains of Tg(FFI) and Tg(CJD) mice, we prepared different brain homogenates from Tg lines expressing 3F4-tagged or untagged mutant PrP at different levels, and co-expressing endogenous WT PrP or not (Table 2). The brain homogenates were inoculated intracerebrally in C57BL/6J mice, and in Tg*a*20 mice that overexpress moPrP at 8X and are highly sensitive to prions [25]. Homogenates from Tg(FFI-K5) and Tg(CJD-A21) mice, expressing 3F4-tagged mutant PrPs, were also inoculated in Tg(WT-$E1^{+/+}$) mice overexpressing WT moPrP with the 3F4 epitope [13], and in Tg(CJD-$G1^{+/+}$) mice, which express low levels of 3F4-tagged D177N/V128 PrP and do not spontaneously become ill [14]. The PrPs expressed by these two lines of mice should be particularly efficient for assaying infectivity because they contain either the 3F4 epitope or the 3F4 epitope and the D177N mutation. All transgenic recipient mice used in this study carried two disrupted *Prnp* alleles, so they did not synthesize endogenous PrP. Brain homogenates from non-Tg/$Prnp^{+/+}$ mice served as negative controls. As positive controls, some host mice were inoculated with the mouse-adapted RML isolate of scrapie that had been previously passaged in WT or Tg(WT-$E1^{+/+}$) mice (RML and RML^{3F4}, respectively) [26]. All mice were observed weekly for the appearance of neurological signs.

None of the animals inoculated with brain homogenates from Tg(FFI) and Tg(CJD) mice, or from negative control mice, developed neurological dysfunction, and all the animals either died from intercurrent illness or were euthanized near the end of their normal lifespan, approximately two years after inoculation (Table 2, lines 2–17, 21–36, 40–49, 53–62). None of the brains from inoculated C57BL/6J, Tg(WT-$E1^{+/+}$) or Tg*a*20 host mice that were subjected to biochemical analysis contained PrP that was detergent-insoluble or that yielded typical or atypical (i.e. 1E4- or SAF84-immunoreactive [27, 28]) PK-resistant fragments. Tg(CJD-$G1^{+/+}$) spontaneously accumulate small amounts of detergent-insoluble PrP in their brains [14], but inoculation with Tg(CJD) or Tg(FFI) brain homogenates did not increase the amount. In contrast, all positive control mice inoculated with RML prions developed scrapie (Table 2, lines 18, 19, 37, 38, 50, 51), and their brains contained PrP that was resistant to high concentrations of PK. Thus, the brains of Tg(FFI) and Tg(CJD) mice did not contain prion infectivity detectable by bioassay.

PMCA Does Not Detect Spontaneously Formed PrP^{Sc} in Tg(FFI) and Tg (CJD) Brains, but the Mutant PrPs Can Be Converted into PrP^{Sc} *in vitro*

To test whether that the brains of Tg(FFI) and Tg(CJD) mice contained prions below the threshold of detection of the bioassay, we subjected the brain homogenates to serial protein misfolding cyclic amplification (PMCA). This allows highly efficient prion replication in a test tube, and is able to amplify the equivalent of a single molecule of PrP^{Sc} [29]. Brain homogenates from Tg(FFI) and Tg(CJD) mice were subjected to serial PMCA with or without a RML seed. RML-seeded PMCA yielded forms of D177N/M128 and D177N/V128 PrPs that were highly PK-resistant (hereafter referred to as $D177N/M128^{RML}$ and $D177N/V128^{RML}$,

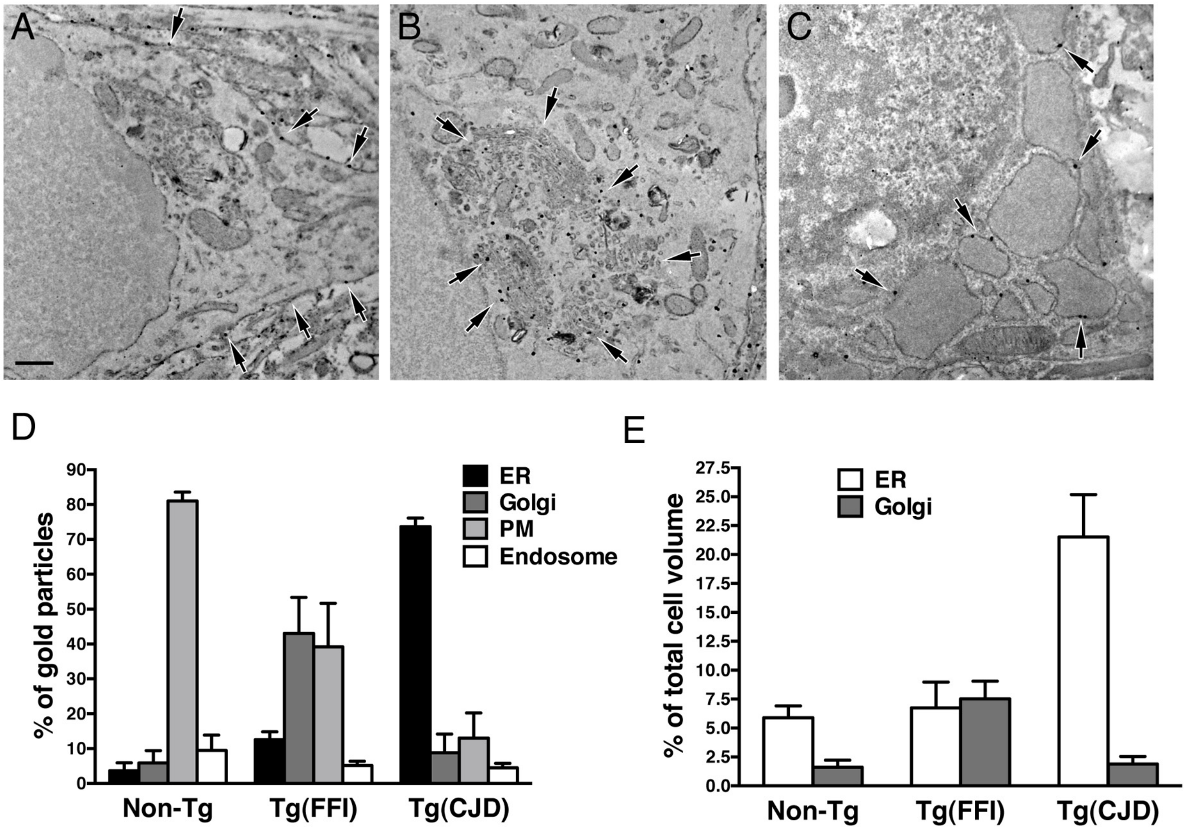

Fig 11. Tg(FFI) and Tg(CJD) neurons show different intracellular PrP accumulations and morphological abnormalities of transport organelles. Cultures of cerebellar granule neurons from non-Tg/*Prnp*$^{+/+}$, Tg(FFI-K5$^{+/-}$)/*Prnp*$^{0/0}$ and Tg(CJD-A21$^{+/-}$)/*Prnp*$^{0/0}$ mice were fixed and labeled with anti-PrP monoclonal antibody 12B2 using the gold-enhance protocol. WT PrP is mostly found at the plasma membrane (A). D177N/M128 PrP is mostly in the Golgi (B), and D177N/V128 PrP is mostly in the ER, whose cisternae appear enlarged and swollen (C). Scale bar = 250 nm in A, B and C. (D) Quantification of gold particles in different cell compartments. PM, plasma membrane. (E) Quantification of ER and Golgi volumes of cultured cerebellar granule neurons. Data are the mean ± SD of at least 10 cells per specimen. Data for non-Tg/*Prnp*$^{+/+}$ and Tg(CJD-A21$^{+/-}$) neurons in D and E are from [14].

doi:10.1371/journal.ppat.1004796.g011

respectively), while the unseeded reactions did not (Fig 12A and 12B), indicating that the brains of Tg(FFI) and Tg(CJD) mice did not contain any spontaneously generated PrP^{Sc}.

To test whether D177N/M128^{RML} and D177N/V128^{RML} corresponded to *bona fide* PrP^{Sc}, the PMCA reactions were intracerebrally inoculated in Tg*a*20 mice. Four out of seven mice inoculated with D177N/M128^{RML}, and five of nine inoculated with D177N/V128^{RML}, had typical PK-resistant PrP^{Sc} in their brains (Fig 12C and 12D), although at the time of death (> 400 days post-inoculation: d.p.i.) they did not have clinical signs of scrapie (for comparison Tg*a*20 mice inoculated with *in vitro* amplified RML developed scrapie at 78 ± 6 d.p.i. and died at 83 ± 6 d.p.i.; mean ± SEM, n = 3). Thus the *in vitro* converted mutant PrPs were able to propagate *in vivo* as authentic prions, but produced subclinical infections, most likely because of differences between the primary structure of the mutant PrP^{Sc} in the infecting inoculum and WT PrP^{C} in the recipient mice [30, 31]. As expected, the brains of Tg*a*20 mice inoculated with the unseeded PMCA reactions did not contain any PK-resistant PrP^{Sc} (Fig 12C and 12D).

Discussion

The present study found that mice carrying a transgene encoding the mouse homologue of the FFI mutation synthesize an abnormal form of PrP in their brains and develop a neurological

Table 2. Transmission assay for infectivity in the brains of spontaneously ill Tg(FFI) and Tg(CJD) mice.

Recipient[a]	Line no.	Inoculum[b]	Time to symptoms[c] (mean days ± SEM)	Time to death[d] (mean days ± SEM)	No. dead/total
C57BL/6J	1	None	>600		0/5
	2	Non-Tg/*Prnp*$^{+/+}$	>540		0/8
	3	Non-Tg/*Prnp*$^{+/+}$	>590		0/7
	4	Tg(FFI-K5$^{+/-}$)/*Prnp*$^{0/0}$	>600		0/6
	5	Tg(FFI-K5$^{+/-}$)/*Prnp*$^{0/0}$	>565		0/10
	6	Tg(FFI-K5$^{+/-}$)/*Prnp*$^{+/0}$	>650		0/7
	7	Tg(FFI-K5$^{+/-}$)/*Prnp*$^{+/+}$	>655		0/5
	8	Tg(FFI-17$^{+/-}$)/*Prnp*$^{+/+}$	>585		0/7
	9	Tg(FFI-26$^{+/-}$)/*Prnp*$^{+/0}$	>555		0/6
	10	Tg(FFI-26$^{+/-}$)/*Prnp*$^{0/0}$	>660		0/9
	11	Tg(CJD-A21$^{+/-}$)/*Prnp*$^{0/0}$	>545		0/9
	12	Tg(CJD-A21$^{+/-}$)/*Prnp*$^{+/0}$	>570		0/9
	13	Tg(CJD-A21$^{+/-}$)/*Prnp*$^{+/0}$	>645		0/8
	14	Tg(CJD-A21$^{+/+}$)/*Prnp*$^{0/0}$	>605		0/11
	15	Tg(CJD-39$^{+/-}$)/*Prnp*$^{0/0}$	>530		0/3
	16	Tg(CJD-66$^{+/-}$)/*Prnp*$^{+/0}$	>580		0/7
	17	Tg(CJD-66$^{+/-}$)/*Prnp*$^{0/0}$	>680		0/7
	18	RML[e]	140 ± 4	153 ± 3	6/6
	19	RML 3F4[f]	171 ± 0	177 ± 1	5/5
Tg*a*20	20	None	>570		0/5
	21	Non-Tg/*Prnp*$^{+/+}$	>495		0/5
	22	Non-Tg/*Prnp*$^{+/+}$	>495		0/4
	23	Tg(FFI-K5$^{+/-}$)/*Prnp*$^{0/0}$	>540		0/4
	24	Tg(FFI-K5$^{+/-}$)/*Prnp*$^{0/0}$	>525		0/6
	25	Tg(FFI-K5$^{+/-}$)/*Prnp*$^{+/0}$	>520		0/7
	26	Tg(FFI-K5$^{+/-}$)/*Prnp*$^{+/+}$	>505		0/10
	27	Tg(FFI-17$^{+/-}$)/*Prnp*$^{+/+}$	>570		0/7
	28	Tg(FFI-26$^{+/-}$)/*Prnp*$^{+/0}$	>495		0/6
	29	Tg(FFI-26$^{+/-}$)/*Prnp*$^{0/0}$	>500		0/8
	30	Tg(CJD-A21$^{+/-}$)/*Prnp*$^{0/0}$	>500		0/7
	31	Tg(CJD-A21$^{+/-}$)/*Prnp*$^{+/0}$	>515		0/9
	32	Tg(CJD-A21$^{+/-}$)/*Prnp*$^{+/0}$	>590		0/7
	33	Tg(CJD-A21$^{+/+}$)/*Prnp*$^{0/0}$	>640		0/5
	34	Tg(CJD-39$^{+/-}$)/*Prnp*$^{0/0}$	>520		0/6
	35	Tg(CJD-66$^{+/-}$)/*Prnp*$^{+/0}$	>545		0/6
	36	Tg(CJD-66$^{+/-}$)/*Prnp*$^{0/0}$	>495		0/6
	37	RML[e]	61 ± 4	66 ± 2	4/4
	38	RML 3F4[f]	85 ± 1	88 ± 2	5/5
Tg(WT-E1$^{+/+}$)/*Prnp*$^{0/0}$	39	None[g]	>540		0/4
	40	Non-Tg/*Prnp*$^{+/+}$	>405		0/5
	41	Non-Tg/*Prnp*$^{+/+}$	>410		0/8
	42	Tg(FFI-K5$^{+/-}$)/*Prnp*$^{0/0}$	>440		0/7
	43	Tg(FFI-K5$^{+/-}$)/*Prnp*$^{0/0}$	>430		0/8
	44	Tg(FFI-K5$^{+/-}$)/*Prnp*$^{+/0}$	>440		0/8
	45	Tg(FFI-K5$^{+/-}$)/*Prnp*$^{+/+}$	>400		0/8
	46	Tg(CJD-A21$^{+/-}$)/*Prnp*$^{0/0}$	>425		0/7

(*Continued*)

Table 2. (*Continued*)

Recipient[a]	Line no.	Inoculum[b]	Time to symptoms[c] (mean days ± SEM)	Time to death[d] (mean days ± SEM)	No. dead/total
	47	Tg(CJD-A21$^{+/-}$)/*Prnp*$^{+/0}$	>440		0/9
	48	Tg(CJD-A21$^{+/-}$)/*Prnp*$^{+/0}$	>450		0/7
	49	Tg(CJD-A21$^{+/+}$)/*Prnp*$^{0/0}$	>450		0/7
	50	RML[e]	217 ± 11[g]	251 ± 26[g]	4/4[g]
	51	RML 3F4[f]	183 ± 4[g]	202 ± 4[g]	5/5[g]
Tg(CJD-G1$^{+/+}$)/*Prnp*$^{0/0}$	52	None	>600		0/6
	53	Non-Tg/*Prnp*$^{+/+}$	>570		0/5
	54	Non-Tg/*Prnp*$^{+/+}$	>550		0/7
	55	Tg(FFI-K5$^{+/-}$)/*Prnp*$^{0/0}$	>570		0/10
	56	Tg(FFI-K5$^{+/-}$)/*Prnp*$^{0/0}$	>525		0/9
	57	Tg(FFI-K5$^{+/-}$)/*Prnp*$^{+/0}$	>590		0/10
	58	Tg(FFI-K5$^{+/-}$)/*Prnp*$^{+/+}$	>515		0/10
	59	Tg(CJD-A21$^{+/-}$)/*Prnp*$^{0/0}$	>630		0/9
	60	Tg(CJD-A21$^{+/-}$)/*Prnp*$^{+/0}$	>530		0/8
	61	Tg(CJD-A21$^{+/-}$)/*Prnp*$^{+/0}$	>510		0/9
	62	Tg(CJD-A21$^{+/+}$)/*Prnp*$^{0/0}$	>535		0/11

[a] Recipient mice were inoculated intracerebrally at 40 to 100 days of age.

[b] Inocula consisted of 10% (w/vol) brain homogenates prepared from mice of the genotypes indicated. Tg(FFI-K5$^{+/-}$) express 3F4-tagged D177N/M128 PrP at ~0.7X; Tg(FFI-17$^{+/-}$) and Tg(FFI-26$^{+/-}$) mice express untagged D177N/M128 PrP at ~4 and ~2X, respectively; Tg(CJD-A21$^{+/-}$) express 3F4-tagged D177N/V128 PrP at ~1X [14]; Tg(CJD-66$^{+/-}$) and Tg(CJD-39$^{+/-}$) express untagged D177N/V128 PrP at ~2 and ~4X, respectively. All Tg(FFI-26), Tg (CJD-A21), Tg(CJD-66) mice, and the Tg(CJD-39) founder, were clinically ill at the time the brain homogenates were prepared.

[c] For mice that remained healthy, the time after inoculation at which the animals were killed to terminate the experiment is given. For the other mice (lines 18, 19, 37, 38, 50 and 51), the time from inoculation to onset of symptoms is given.

[d] Time from inoculation to death. Mice that died of nonneurological intercurrent illness are excluded.

[e] RML prions passaged repeatedly in CD1 mice, then once in 129 S2/SvHsd mice.

[f] RML prions passaged repeatedly in CD1 mice, then twice in Tg(WT-E1$^{+/+}$)/*Prnp*$^{0/0}$ mice.

[g] Data are from reference [26].

doi:10.1371/journal.ppat.1004796.t002

illness causing abnormal sleep-wake behavior with dramatic disruption of sleep architecture and circadian organization. The sleep abnormalities in Tg(FFI) mice are different from those of Tg(CJD) mice in which, like in CJD^{178}, the sleep-wake organization is partially preserved [14]. Phenotypic differences are also seen at cellular level, with neurons in Tg(FFI) and Tg (CJD) mice showing different PrP accumulations in the secretory pathway and morphological alterations of transport organelles. The Tg(FFI) and Tg(CJD) diseases could not be transmitted to other mice by intracerebral inoculation, and no PrP^{Sc} could be amplified from brain homogenates of the mutant mice by unseeded PMCA. Thus, the mutant PrPs expressed in these mice are pathogenic but not infectious. Our results suggest a neurotoxic modality involving mutant PrP accumulation in transport organelles, which may help explain the phenotypic heterogeneity of genetic prion diseases.

Sleep Disturbances in Tg(FFI) Mice Are Reminiscent of Those in FFI Patients and Different from Those of Tg(CJD) Mice

The main EEG and sleep alterations of FFI patients [32] are also seen in Tg(FFI)/*Prnp*$^{0/0}$ mice. Longitudinal 24-h monitoring and spectral EEG analysis show a marked reduction in sleep

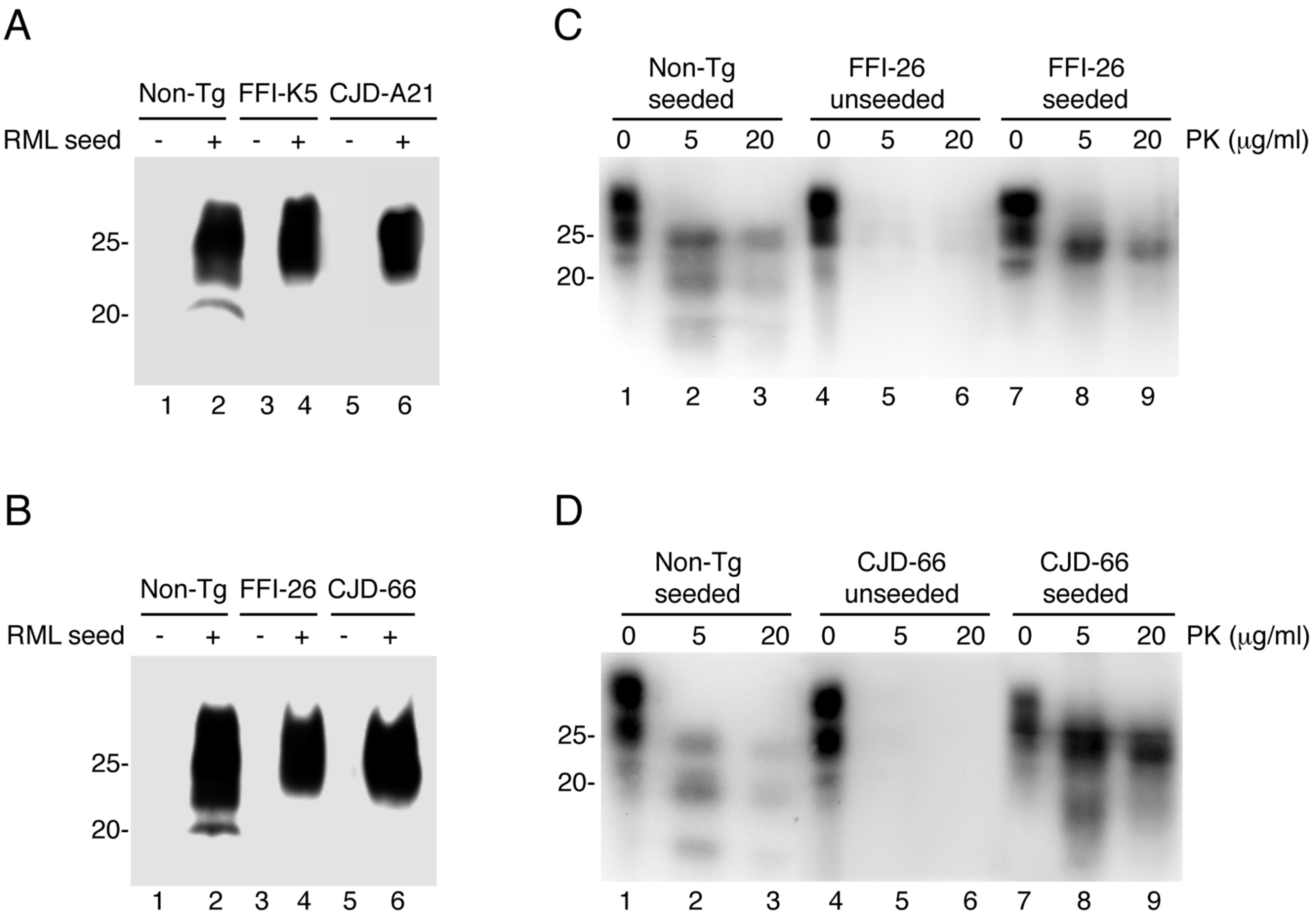

Fig 12. Serial PMCA does not detect spontaneously formed PrP^{Sc} in Tg(FFI) and Tg(CJD) brains, but the mutant PrPs can be converted into PrP^{Sc} *in vitro*. (A and B) Homogenates of 3–4 pooled brains of C57BL/6J (Non-Tg), Tg(FFI-K5$^{+/-}$)/*Prnp*$^{0/0}$, Tg(FFI-26$^{+/-}$)/*Prnp*$^{0/0}$, Tg(CJD-A21$^{+/-}$)/*Prnp*$^{0/0}$ and Tg (CJD-66$^{+/-}$)/*Prnp*$^{0/0}$ mice were subjected to serial rounds of PMCA without (-) or with (+) a PrP^{Sc} inoculum (RML seed). Ten rounds of 48 PMCA cycles were done, and the samples were digested with 80 μg/ml PK before Western blot with anti-PrP antibody SAF84. (C and D) Brain lysates from Tg*a*20 mice inoculated with the reaction products of 17 rounds of PMCA (unseeded or seeded with RML, as indicated) were incubated with 0–20 μg of PK for 30 min at 37°C, and PrP was visualized by Western blotting using antibody 12B2. The undigested samples (0 μg/ml PK) represent 10 μg of protein, and the other samples 50 μg. Mice were killed at 86 (Non-Tg seeded), 558 (FFI-26 unseeded and CJD-66 seeded), 573 (FFI-26 seeded) and 611 (CJD-66 unseeded) days post-inoculation.

doi:10.1371/journal.ppat.1004796.g012

spindles in FFI patients [32], and sleep spindle density is reduced by 75–80% in Tg(FFI)/*Prnp*$^{0/0}$ mice compared to non-Tg controls. Although there is no change in the amount of NREM sleep, SWA during NREM sleep (a measure of sleep drive and depth [17]) is significantly reduced in Tg(FFI)/*Prnp*$^{0/0}$ mice, like in FFI patients [32].

Tg(FFI)/*Prnp*$^{0/0}$ mice also present a profound disruption of sleep continuity and organization. They have a larger number of transitions between the different behavioral states than non-Tg mice. In addition, in Tg(FFI)/*Prnp*$^{0/0}$ mice approximately one fourth of REM sleep episodes starts directly from wakefulness, instead of being preceded by NREM sleep as normally occurs; this is reminiscent of the sudden-onset episodes of REM sleep that intrude into wakefulness in FFI patients [32, 33]. The amount of REM sleep is also significantly decreased in Tg (FFI)/*Prnp*$^{0/0}$ mice, as often happens in FFI patients at a late stage of disease [33]. Finally, Tg (FFI)/*Prnp*$^{0/0}$ mice show an abnormal EEG pattern during REM sleep, with a significant reduction in theta activity. Cyclic organization of sleep and circadian motor rhythmicity are lost in

FFI patients [32], and we found that the circadian organization of sleep and motor activity was lost in Tg(FFI)/*Prnp*$^{0/0}$ mice.

Bursts of quasi-periodic sharp waves at 0.5–2 Hz (similar to the periodic sharp-wave complexes in the EEG activity of CJD patients) may appear in advanced stages of the long-evolution cases of FFI [32–34]. Bursts of high-voltage polyphasic complexes, similar to those described in Tg(CJD) mice [14], were detected in the EEG of Tg(FFI)/*Prnp*$^{0/0}$ mice.

Interestingly, some sleep abnormalities were attenuated in Tg(FFI)/*Prnp*$^{+/0}$ mice co-expressing endogenous WT PrP. For example the amount of REM sleep and circadian organization of sleep and motor rhythms are normal in these mice but they respond abnormally to sleep deprivation, not fully compensating the loss. Moreover, during the light phase, they show more sleep fragmentation than non-Tg/*Prnp*$^{+/+}$ mice, and have less sleep spindle density and abnormal EEG activity, although less than Tg(FFI)/*Prnp*$^{0/0}$ mice. PrP^C has been assigned a role in promoting sleep continuity and circadian rhythmicity [35]. Our observation that re-introduction of one WT PrP allele confers some protection against the sleep and EEG changes induced by the D177N/M128 mutation suggests that functional loss of PrP^C may contribute to sleep disruption in FFI mice. It would be interesting to see whether co-expression of WT PrP at a level comparable to that of transgenic mutant PrP completely reverses the sleep phenotype.

The alterations in sleep and circadian rhythmicity in Tg(FFI) mice differ significantly from those in Tg(CJD) mice modeling CJD^{178} [14]. Whereas in Tg(FFI) mice the circadian organization of sleep and motor activity is lost, sleep is fragmented, and theta activity (the EEG hallmark of rodent REM sleep) is reduced, in Tg(CJD) mice the circadian organization of sleep, its continuity and its EEG patterns are not altered [14]. Thus Tg(FFI) and Tg(CJD) mice recapitulate specific phenotypic features of the corresponding human diseases.

Other Pathological Features

Ataxia and other motor symptoms, such as myoclonus, tremor, dysarthria and pyramidal impairment, are clinical features of FFI, which in some cases can be the earliest and most marked neurological signs [36–40]. We found that Tg(FFI) mice developed ataxia and sensorimotor deficits, such as inability to climb on a vertical grill, poor performance on a rotating rod, kyphosis and foot clasp reflex. The time of onset and rate of progression of these symptoms is directly correlated with transgene dosage. Tg(FFI) mice with mutant PrP expression below the level of endogenous PrP remain healthy throughout their lifetime. Mild neurological signs develop in ~60% of hemizygous Tg(FFI-10) mice expressing mutant PrP at the endogenous level, and these animals live as long as nontransgenic controls, similar to knockin ki-3F4-FFI mice in which 3F4-tagged D177N/M128 PrP is under the control of the endogenous *Prnp* promoter [41]. In contrast, all Tg(FFI) mice expressing mutant PrP twice the endogenous level or more develop progressive and invariably fatal neurological disease. Transgenic mice expressing WT PrP up to seven times the endogenous level remain healthy during their lifetime [13, 42], and have normal sleep-wake behavior [14], strongly indicating that the Tg(FFI) phenotype is not a mere consequence of overexpression, but is due to the D177N/M128 mutation.

In contrast to the mitigating effect on sleep abnormalities, co-expression of WT PrP does not significantly modify the time of onset and progression of motor dysfunction or prolong survival of Tg(FFI) mice, consistent with the dominant mode of inheritance of FFI and with similar observations in other mutant PrP mice [14, 19, 43]. Thus, WT PrP influences only some aspects of the Tg(FFI) phenotype, perhaps those that are more dependent on a physiological function of PrP in neuronal excitability [44].

Disturbances of attention and memory, difficulties with the temporal ordering of events and spatial disorientation are early signs of FFI, which usually appear before ataxia and other

motor deficits [32, 45]. Tg(FFI) mice have significant alterations in recognition and spatial working memory, detectable in the object recognition test and the eight-arm radial maze before the onset of motor dysfunction. Thus salient motor and cognitive aspects of the clinical picture of FFI are recapitulated in transgenic mice.

Tg(FFI) mice also show brain abnormalities reminiscent of human FFI. Thalamic degeneration is the most marked neuropathological change in FFI patients, who frequently also have degenerated cerebella [36, 40, 46], and reduced thalamic and cerebellar volumes were detected by MRI in Tg(FFI) mice in the advanced stage of disease.

Although PrP^{Sc} accumulates to lower levels in FFI than in other human prion diseases, widespread protease-resistant PrP deposits can be detected by immunohistochemistry in the CNS of FFI patients, especially in cases of long duration [47]. These include fine-granular synaptic-type and focal PrP deposits, patchy and strip-like immunoreactive profiles, as well as intravacuolar and cytoplasmic PrP accumulations in neurons [40]. Synaptic-type, dot-like and small round PrP profiles, as well as immunoreactive fiber tracts and intraneuronal PrP deposits, reminiscent of those in FFI patients, are detected in the brains of Tg(FFI) mice. Moderate gliosis is seen in several brain regions of Tg(FFI) mice, including the external layers of the cerebral cortex and the periaqueductal gray, similar to findings in FFI patients where gliosis has a bilaminar accentuation in the cerebral cortex and is often detected in the periaqueductal gray [40, 47].

D177N PrP Is Pathogenic but Not Infectious

Mutant PrP in the brains of Tg(FFI) and Tg(CJD) mice displays several biochemical characteristics reminiscent of PrP^{Sc}, including insolubility in non-denaturing detergents, low PK resistance and reactivity with PrP^{Sc}-directed antibodies [14, 48, 49 and this study], raising the possibility that the protein may have spontaneously acquired an infectious conformation. However, we found that brain homogenates from Tg(FFI) and Tg(CJD) mice had no detectable infectivity when inoculated into different transgenic and nontransgenic hosts. We also found no amplification of PrP^{Sc} in unseeded PMCA reactions, ruling out the possibility that the inocula contained prions below the threshold of detection by our bioassay. Thus mutant PrP in the brains of Tg(FFI) and Tg(CJD) mice is misfolded and pathogenic but unable to propagate its abnormal conformation, and is therefore fundamentally different from PrP^{Sc}. However, it is not intrinsically refractory to PrP^{Sc} conversion since it acquires an infectious conformation when subjected to PMCA in the presence of a RML seed.

Our results are different from those of Jackson et al., who reported the emergence of spontaneous prion infectivity in knockin ki-3F4-FFI mice expressing 3F4-tagged D177N/M128 PrP from the endogenous *Prnp* locus [41]. Ki-3F4-FFI mice did not die prematurely, but developed behavioral abnormalities in old age, similar to our hemizygous Tg(FFI-10) mice expressing mutant PrP at the endogenous level. Intracerebral inoculation of brain homogenates from sick ki-3F4-FFI mice induced neurological disease in ki-3F4-WT mice expressing 3F4-tagged WT PrP, and in Tg*a*20 mice, but not in nontransgenic mice [41], arguing that homology between mutant PrP and the recipient's PrP^{C} at residues 108 and 111 (which constitute the 3F4 epitope), or overexpression of untagged PrP by the recipient mice, is required for disease transmission.

In contrast with this, we found that the Tg(FFI) disease could not be transmitted to other mice, despite homology between mutant and the recipient's PrP, and/or PrP overexpression in the recipient mice. The reason for this difference is not clear. It was suggested that mutant PrP needs to be targeted to the *Prnp* locus to generate a transmissible agent spontaneously [41]. However, prions emerged spontaneously even in mice with randomly integrated transgenes, including those constructed using the half-genomic *Prnp* vector used in our study [21, 50–53]. In addition, a gene targeting approach identical to the one used by Jackson et al. was employed

to generate P101L mice expressing the mouse homolog of the P102L mutation linked to GSS [54], and these mice did not develop a transmissible prion disease [54]. Thus, replacing the endogenous PrP coding sequence is neither necessary nor sufficient for *de novo* prion generation.

Ki-3F4-FFI mice have a mixed 129/Ola X C57BL/6N genetic background, whereas our Tg (FFI) mice are C57BL/6J x CBA/J hybrids backcrossed with C57BL/6J mice. It is possible that the genetic makeup of ki-3F4-FFI mice favors generation of prion infectivity, for example because co-factors that might promote PrP^{Sc} formation [55, 56] are selectively expressed or enriched in these animals. However, prion infectivity developed *de novo* even in Tg PrP mice with C57BL/6J X 129S5 [50], C57BL/6 X FVB [52], C57BL/6J X CBA/J X 129/Ola [21] and FVB [51, 53] backgrounds, suggesting that a specific allelic composition is not required.

Mutant PrP in the brains of ki-3F4-FFI mice does not show the typical biochemical attributes of PrP^{Sc}, suggesting that ki-3F4-FFI mice bear an unconventional prion strain [41]. However, small amounts of protease resistant PrP were detected when 20 mg of total brain homogenates from two-year-old ki-3F4-FFI mice was PK digested and concentrated by trichloroacetic acid precipitation [41]. It would be interesting to see whether this form of the protein can be amplified by PMCA. It would also be interesting to compare the conformations of D177N/M128 PrP molecules extracted from the brains of ki-3F4-FFI and our Tg(FFI) mice, because this may shed light on the structural features that enable mutant PrP to self-replicate. In this regard, we have found that infectious and non-infectious forms of PG14 PrP are structurally related but differ in their oligomeric state and degree of PK resistance [26, 48, 57].

Different prion diseases are thought to be enciphered in distinctive conformations of the PrP^{Sc} molecule (prion strains) [24, 58]. Our observation that Tg(FFI) and Tg(CJD) mice recapitulate specific features of the respective human disorders without developing prion infectivity—and the same holds true for Tg(PG14) mice and other mouse models of GSS [20, 26, 59 and J. Mastrianni, personal communication]—suggests that the disease-encoding properties of mutant PrP are enciphered in misfolded conformations of the protein that are toxic but not infectious [60, 61]. We recently obtained evidence that the mutant PrPs extracted from the brains of Tg(FFI), Tg(CJD) and Tg(PG14) mice have different structures, indicating that they carry enough conformational diversity to encode different diseases [49].

Our observation that the pathogenicity of misfolded PrP does not depend on its ability to self-replicate has implications for other neurodegenerative diseases associated with protein misfolding. There is evidence that aggregated proteins such as amyloid-β in AD, tau in the tauopathies, and α-synuclein in PD, are able to spread in a prion-like manner when inoculated into the mouse brain [62]. However, the mechanism linking spread of protein misfolding and neurodegeneration in these diseases is not clear [63]. Our results are consistent with a dissociation between the toxic and propagating PrP species [60, 61], and suggest that there may be a separation between spread of misfolded proteins and neurotoxicity also in other neurodegenerative diseases.

Impaired Trafficking of Mutant PrP and Its Potential Role in Phenotypic Expression of Disease

We have reported that mouse PrP molecules carrying the D177N mutation are delayed in their biosynthetic maturation and are partially retained in transport organelles of the secretory pathway [14, 64–66]. In the present study we found that D177N/M128 PrP accumulates preferentially in the Golgi of Tg(FFI) neurons, and this is associated with enlargement of the Golgi. In contrast, D177N/V128 PrP is mostly found in the ER of Tg(CJD) neurons, and this organelle appears swollen and electrondense [14]. Thus the two polymorphic variants tend to accumulate in different intracellular compartments, causing different morphological alterations of transport organelles. The intracellular accumulation of D177N/M128 and D177N/V128 PrPs also differed

in transfected cells [65, 67], and may reflect the way these mutants acquire abnormal conformations and aggregate during secretory transport [68]. There is evidence, in fact, that the M/V 129 polymorphism influences the kinetics of misfolding and oligomerization of D178N PrP [11], and oligomerization and intracellular retention of this mutant are closely related [69].

We previously found that ER retention of PG14 and D177N/V128 PrPs impairs the transport of VGCCs to presynaptic terminals of CGNs, due to a physical interaction of PrP with the auxiliary $\alpha_2\delta$-1 subunit of the channel [15]. Since artificial targeting of PrP to the Golgi results in intracellular retention of $\alpha_2\delta$-1 [15], D177N/M128 may also impair VGCC transport and function in CGNs, contributing to the motor dysfunction of Tg(FFI), as in Tg(PG14) and Tg (CJD) mice. However, there could be other PrP^C-interacting proteins whose cellular trafficking and synaptic targeting may be affected differently by the different mutants, potentially triggering specific neurotoxic effects [70]. PrP^C interacts physically with the NR1 and NR2D subunits of N-methyl-D-aspartate (NMDA) and the GluA1 and GluA2 subunits of α-amino-3-hydroxy-5-methyl-4-isoxazolepropionic acid (AMPA) receptors, and these interactions are important for normal neuronal physiology and survival [71–73]. Interestingly, the assembly and trafficking of these receptors are finely tuned in the ER and Golgi [74, 75]. Our preliminary observations indicate that mutant PrPs that accumulate in different intracellular organelles affect NMDA and AMPA receptor trafficking in different ways. Moreover, different mutants interact differently with receptor subunit isoforms expressed in functionally distinct neurons of the brain [73]. Thus, different mutant PrPs may have different effects on the function and survival of different neurons—hence on the clinical presentation of disease—depending on where in the secretory pathway they preferentially localize, and how this interferes with the transport of the molecules they interact with [70].

In summary, we have generated transgenic mice that model essential aspects of FFI, and differ from analogous mice expressing the CJD^{178} mutation. Disease-specific features are seen in independently generated Tg lines with different copies of integrated transgene and PrP expression levels, strongly indicating that they are encoded by mutant PrP, rather than non-specific effects of random transgenesis. Tg(FFI) mice may be useful for investigating the pathophysiology of sleep in FFI and for testing potential therapies for this devastating disorder. Comparative studies of Tg(FFI) and Tg(CJD) mice may provide important information on the molecular mechanisms responsible for the phenotypic heterogeneity associated with the polymorphic variants of the PrP D178N mutation.

Materials and Methods

Transgenic Mice

The cDNAs encoding mouse PrP derived from the *Prnp^a* allele, containing the D177N/M128 substitution with or without the 3F4 epitope tag, were ligated into the MoPrP. Xho vector, which contains a 12 kb fragment of *Prnp*, including the promoter and intron 1, and drives the expression of transgenic PrP in a tissue pattern similar to that of the endogenous protein [14, 76]. Recombinant plasmids were selected by PCR screening and restriction analysis, and their identity confirmed by sequencing the entire coding region [13]. The transgene was excised by NotI digestion and injected into the pronuclei of fertilized eggs from an F_2 cross of C57BL/6J x CBA/J F_1 parental mice. Transgenic founders were bred with an inbred colony of Zürich I *Prnp*$^{0/0}$ mice [77] with a pure C57BL/6J background (C57BL/6J/*Prnp*$^{0/0}$; European Mouse Mutant Archive, Monterotondo, Rome, Italy; EM:01723). The status of the *Prnp* gene and the presence of the transgene were determined by PCR, and the zygosity of the transgene by RT-PCR [13]. Production of transgenic Tg(WT-E1$^{+/-}$) mice, expressing wild-type PrP tagged with an epitope for monoclonal antibody 3F4 at approximately 2X, and Tg(CJD-A21$^{+/-}$) and

Tg(CJD-G1$^{+/+}$) mice expressing 3F4-tagged D177N/V128 PrP at ~1X and ~0.3X respectively, has already been reported [13, 14]. Tg(CJD-66$^{+/-}$) and Tg(CJD-39$^{+/-}$) mice, which express un-tagged D177N/V128 PrP at ~2X and ~4X respectively, were generated as described above. They develop a CJD-like neurological syndrome like Tg(CJD-A21) mice [14], which will be described in details elsewhere. All transgenic lines used in this study were backcrossed for at least ten generations with C57BL/6J/*Prnp*$^{0/0}$ mice, with the exception of Tg*a*20 (EM:00181) [25], Tg (CJD-G1) (EM:06144) and Tg(WT-E1$^{+/+}$) [14] mice which were maintained as inbred hybrid colonies. For some experiments the *Prnp* allele was re-introduced by breeding transgenic mice with C57BL/6J (Harlan Laboratories).

Ethics Statement

Procedures involving animals and their care were conducted in conformity with the institutional guidelines at the IRCCS—Mario Negri Institute for Pharmacological Research in compliance with national (D.lgs 26/2014; Authorization n. 19/2008-A issued March 6, 2008 by Ministry of Health) and international laws and policies (EEC Council Directive 2010/63/UE; the NIH Guide for the Care and Use of Laboratory Animals, 2011 edition). They were reviewed and approved by the Mario Negri Institute Animal Care and Use Committee that includes ad hoc members for ethical issues (18/01-D and 18/02-D), and by the Italian Ministry of Health (Decreto nr 62/2012-B and 63/2012-B). Animal facilities meet international standards and are regularly checked by a certified veterinarian who is responsible for health monitoring, animal welfare supervision, experimental protocols and review of procedures.

Biochemical Analyses

Assays of detergent insolubility and proteinase K resistance were done as described [13]. Western blots were developed with monoclonal anti-PrP antibodies 12B2 (Central Veterinary Institute, Wageningen, NL), 1E4 (Cell Sciences), or SAF84 (Spi Bio).

Sleep-Wake Behavior and Electroencephalographic (EEG) Activity

We investigated EEG and sleep patterns in eight non-Tg/*Prnp*$^{+/+}$, ten non-Tg/*Prnp*$^{0/0}$, nine Tg (FFI-26$^{+/-}$)/*Prnp*$^{0/0}$, and eight Tg(FFI-26$^{+/-}$)/*Prnp*$^{+/0}$ mice aged between 332 and 468 days of age. Mice were anesthetized and instrumented for chronic EEG recording according to standard techniques [78]. They were individually housed in standard cages with food and water ad libitum, and allowed at least ten days to recover from surgery and adapt to the recording conditions. Cages were kept in sound-attenuated rooms at a constant temperature (26 ± 1°C), with a 12/12-hour light-dark cycle. Gross body activity was detected using an infrared sensor housed in an observation unit that also contained a camera (BioBserve GmbH, Bonn, Germany), allowing undisturbed monitoring of the animals' behavior. Movements detected by the infrared sensor were converted to a voltage output. The conditioned EEG signal and the voltage output from the infrared sensor were digitized and collected using custom software (M. R. Opp, University of Michigan). For investigations in undisturbed conditions, EEG signals and gross body activity were recorded for 24 h (starting at the beginning of the light phase) and used for polygraphic determination of vigilance. The animals were not handled starting from 48 h before the recording session.

For investigations of the effects of sleep deprivation, mice were sleep-deprived by gentle handling for the first six hours of the light phase, then allowed to behave and sleep freely for the next 18 hours (i. e. for the second six hours of the light phase followed by the12 hours of the dark phase). Two mice of different genotypes were always randomly matched and recorded simultaneously. The order of recording of mice of the different lines was also randomized.

Postacquisition determination of vigilance was done according to standard criteria [78]. An investigator (F.D.G.) blind to the strain visually scored 12-s epochs. EEG power densities were obtained for each animal and each behavioral state by Fourier transform for each artifact-free 12-s scoring epoch for the frequency range 0.5–20 Hz. Values in the 0.5–4.0 Hz (delta) frequency range were collapsed and integrated for 12-s epochs, and used as measures of SWA during NREM sleep. EEG recordings during the light portion of the light-dark phase were also band-pass filtered (10–13 Hz, 4th order Chebyschev type II filter), and NREM sleep spindles were visually identified. Spindle density was obtained (number of spindles/hour of NREM sleep). Statistical analysis was done as described in the Results section or in the legends to tables and figures.

Clinical Analysis of Mice

Mice were observed weekly for signs of neurological dysfunction, according to a set of objective criteria [13]. Onset of disease was scored as the time at which at least two neurological signs were observed, out of foot-clasp reflex, kyphosis, unbalanced body posture, inability to walk on a horizontal metal grid, and to remain on a vertical grid for at least 30 s. The accelerating Rotarod 7650 model (Ugo Basile) was used: mice were first trained three times the week before official testing. They were positioned on the rotating bar and allowed to become acquainted with the environment for 30 s. The rod motor was started at an initial setting of 7 r.p.m. and accelerated to 40 r.p.m. at a constant rate of 0.11 r.p.m./s for a maximum of 300 s. Performance was scored as latency to fall, in seconds. Animals were given three trials, and the average was used for statistical analysis.

Radial Maze

Spatial working memory was measured using an eight-arm radial maze made of grey plastic with a Plexiglas lid. The arms radiating from an octagonal central arena with a diameter of 12 cm, were 30 cm long, 5 cm wide and 4 cm high. Several extra-maze visual cues surrounded the apparatus. Starting one week before testing, the mice were water-deprived by allowing them water for only one hour a day. One day before starting the task schedule, a habituation trial was run. The mice were placed in the center of the maze and left free to explore the environment for 5 minutes. The next day the arms of the radial maze were baited with 50 μl of water. Animals were placed in the center of the maze and the arm-entry sequence was recorded. The task ended once all eight arms of the maze had been visited or after a maximum of 16 trials, whichever came first. Repeated entry into an arm that had already been visited constituted an error. The number of errors and the latency to complete the test were recorded manually by an operator (I.B.) blind to the experimental groups. Animals were tested for 16 consecutive days.

Object Recognition

Mice were tested in an open-square grey arena (40 x 40 cm), 30 cm high, with the floor divided into 25 squares by black lines. The following objects were used: a black plastic cylinder (4 x 5 cm), a glass vial with a white cap (3 x 6 cm) and a metal cube (3 x 5 cm). The task started with a habituation trial during which the animals were placed in the empty arena for 5 minutes and their movements were recorded as the number of line-crossings. The next day, mice were placed in the same arena containing two identical objects (familiarization phase). Exploration was recorded in a 10-minute trial by an investigator (C.B.) blinded to the experimental group. Sniffing, touching and stretching the head toward the object at a distance of not more than 2 cm were scored as object investigation. Twenty-four hours later (test phase) mice were placed in the arena containing two objects: one identical to one of the objects presented during the familiarization phase (familiar object), and a new, different one (novel object), and the time

spent exploring the two objects was recorded for 10 min. Memory was expressed as a discrimination index, i.e. the time spent exploring the novel object minus the time spent exploring the familiar object, divided by the total time spent exploring both objects; the higher the discrimination index, the better the performance.

MRI

Animals were anesthetized with 1% isoflurane in a 30:70% O_2:N_2O gas mixture and imaged in a horizontal bore 7-Tesla USR preclinical MRI system (BioSpec 70/30, Bruker BioSpin, Germany) with a shielded gradient insert (BGA 12, 400 mT/m; rise time, 110 us). A 72-mm birdcage resonator for RF transmission, and a 10-mm diameter single-loop receiver coil were used to receive the signal. 3D T2-weighted anatomical images of the mouse brain were acquired with the following parameters: TR 2500 ms, TE 50 ms, RARE factor 16, FOV 3 x 1.5 x 1.5 cm, Matrix 256 x 102 x 102, voxel 0.147 x 0.117 x 0.147. The scan time was approximately 25 min. The volumes of the whole brain and individual brain areas (frontal cortex, hippocampus, thalamus, striatum and cerebellum) were quantified manually using Fiji software [79], after a rigid body registration (6 dof) to a reference image to avoid bias due to bad head positioning.

Histology

Mice were euthanized by CO_2 inhalation, brains were removed and fixed in Alcolin (Diapath) or Carnoy's fixative (ethanol, chloroform, acetic acid, 6:3:1), dehydrated in graded ethanol solutions, cleared in xylene, and embedded in paraffin. Serial sections (8 mm thick) were cut and stained with hematoxylin and eosin, Nissl, or thioflavin S. For PrP immunohistochemistry, sections were incubated with PK (2 μg/ml in H_2O) for 1 h at room temperature, and exposed to guanidine thyocianate (3M in H_2O) for 30 min [80]. PK-resistant PrP was detected with monoclonal antibody 12B2 (1:2000), using the ARK kit (Dako), with 3,3' diaminobenzidine (DAB) as chromogen.

For glial fibrillary acidic protein (GFAP) and ionized calcium binding adapter molecule 1 (Iba1) immunohistochemistry, mice were deeply anesthetized by intreperitoneal injection of 100 mg/kg ketamine hydrochloride and 1 mg/kg medetomidine hydrochloride (Alcyon), and perfused through the ascending aorta with phosphate buffered saline (PBS, 0.05 M; pH 7.4) followed by 4% paraformaldehyde (PFA) in PBS. Brains were removed, post-fixed, cryoprotected and frozen at -80°C. Sections were cut using a Leica cryostat and incubated for 1 h at RT with 10% normal goat serum (NGS), 0.3% Triton X-100 in PBS 0.1M, pH 7.4, then overnight at 4°C with mouse monoclonal anti-GFAP antibody (Millipore, 1:2500) or rabbit polyclonal anti-Iba1 (Wako Reagent, 1:1000), followed by visualization with the Vectastain ABC kit (Vector), using DAB as chromogen.

Electron Microscopy

One 503-day-old Tg(WT-E1$^{+/-}$)/*Prnp*$^{0/0}$ mouse, one 269-day-old Tg(WT-E1$^{+/+}$)/*Prnp*$^{0/0}$ mouse, two non-Tg/*Prnp*$^{0/0}$ mice aged 208 and 374 days, four non-Tg/*Prnp*$^{+/+}$ mice 152, 268, 280 and 392 days old, one 302 and two 367 days old Tg(FFI-26$^{+/-}$)/*Prnp*$^{0/0}$ mice, one 444 days old Tg(FFI-26$^{+/-}$)/*Prnp*$^{+/0}$ and two Tg(FFI-10$^{+/-}$)/*Prnp*$^{0/0}$ mice aged 331 and 379 days were analyzed by electron microscopy. Mice were deeply anesthetized and perfused through the ascending aorta with phosphate buffered saline (PBS, 0.1 M; pH 7.4) followed by 4% paraformaldehyde (PFA) and 2.5% glutaraldehyde in PBS. The brain was excised, cut along the sagittal plane with a razor blade, and postfixed in 3% glutaraldehyde in PBS then for 2 h in OsO_4. After dehydration in graded series of ethanol, tissue samples were cleared in propylene oxide, embedded in epoxy medium (Epon 812 Fluka) and polymerized at 60°C for 72 h. From

each sample, one semithin section (1 μm) was cut with a Leica EM UC6 ultramicrotome and mounted on glass slides for light microscopic inspection to identify the area of interest. Ultrathin sections (70 nm thick) were obtained, counterstained with uranyl acetate and lead citrate, and examined with an Energy Filter Transmission Electron Microscope (EFTEM, ZEISS LIBRA® 120) equipped with a YAG scintillator slow-scan CCD camera. Lipofuscin granules and cytoplasm of neurons were manually outlined, and areas were calculated with image analysis software (iTem, Olympus). The sum of the areas occupied by lipofuscin in each neuron was expressed as a percentage of the cytoplasmic area. Electron tomography and three-dimensional reconstruction was done as described [81]. Immuno-electron microscopy of PrP in cultured cerebellar granule neurons, quantification of gold particles in the different compartments of the secretory pathway, and analysis of total cell, ER and Golgi volumes, were as described [14].

Protein Misfolding Cyclic Amplification

The detailed protocol for serial PMCA has been published elsewhere [82]. Briefly, 50 μl of 10% brain homogenate were loaded into 0.2-ml PCR tubes and positioned on an adaptor on the plate holder of a S-700MPX sonicator (QSonica, Newtown, CT, USA). Each PMCA cycle consisted of 30 min incubation at 37°C followed by a 20 s sonication pulse at a potency of 70–90. Samples were incubated without shaking immersed in the water of the sonicator bath. After a round of 48 cycles, a 10 μl portion of the amplified material was diluted into 90 μl of brain homogenate and a new round of 48 PMCA cycles was done. This procedure was repeated 10 times. For seeded PMCA, RML-derived, *in vitro* amplified PrP^{Sc} was added to the brain homogenates before PMCA. Samples were digested with 80 μg/ml of PK for 1h at 42ºC and analyzed by Western blot with anti-PrP antibody SAF84. To produce enough material for transmission studies, and to rule out the possibility of residual RML in the seeded PMCA inocula, the reactions were run for seven additional rounds (final dilution 10^{-20}) [29].

Transmission Studies

Ten-percent (w/v) homogenates of mouse brain were prepared in PBS and cleared by centrifugation at 900 x g for 5 min. Serial PMCA reactions were diluted 1:10 in PBS before inoculation. 25 μl of the cleared homogenate, or diluted PMCA reactions, was injected intracerebrally into the right parietal lobe of recipient mice using a 25-gauge needle.

Supporting Information

S1 Fig. Detergent-Insoluble and PK-Resistant PrP Is Detectable in Presymptomatic Mice. (A) Brain lysates from Tg(WT-$E1^{+/-}$)/$Prnp^{0/0}$, Tg(FFI-$26^{+/-}$)/$Prnp^{0/0}$ and Tg(CJD-$66^{+/-}$)/$Prnp^{0/0}$ mice of the indicated ages were ultracentrifuged at 186,000 x g for 40 min, and PrP in the supernatants (S lanes) and pellets (P lanes) was analyzed by Western blotting using the 12B2 antibody. (B) Brain lysates were incubated with 0–2 μg of PK for 30 min at 37°C, and PrP was visualized by Western blotting using antibody 12B2. The undigested samples (0 μg/ml PK) represent 25 μg of protein, and the other samples 100 μg.
(TIF)

S2 Fig. Neurological Symptoms in Tg(FFI) mice. (A) The Tg(FFI-$28^{+/-}$)/$Prnp^{+/+}$ founder at 443 days of age shows kyphosis (hunchback position) and abnormal gait with extension of the hind limbs. (B) When suspended by its tail a Tg(FFI-$10^{+/+}$)/$Prnp^{0/0}$ mouse aged 702 days tightly clasps its hind limbs, whereas a Tg(FFI-$10^{+/-}$)/$Prnp^{0/0}$ littermate (C) splays its limbs. (D) A Tg(FFI-$26^{+/-}$)/$Prnp^{0/0}$ mouse at 418 days is incapable of deambulating on a metal grill.
(TIF)

S3 Fig. Tg(FFI) Mice Show Proliferation of Astrocytes and Microglia in Several Brain Regions. (A-F) Brain sections from Tg(FFI-$26^{+/-}$)/*Prnp*$^{0/0}$ and non-Tg littermates aged 473 (A and B, hippocampus), 204 (C and D, cerebral cortex) and 198 (E and F, cerebellum) days, were stained with anti-glial fibrillar acidic protein (GFAP) antibody. Immunostaining revealed marked astrocytosis in Tg(FFI) but not in non-Tg mice. (G-L) Immunostaining with anti-ionized calcium binding adapter molecule 1 (Iba1) shows marked microgliosis in the hippocampus (G-J), and periaqueductal gray (K and L) of Tg(FFI-$26^{+/-}$)/*Prnp*$^{0/0}$ mice at 533 (H and L) or 473 (J) days compared to non-Tg/*Prnp*$^{0/0}$ littermates. Results were similar with the anti-CD11b antibody. Scale bars = 100 μm in A and B, 50 μm in C, D, I and J, 200 μm in E and F, and 500 μm in G, H, K and L.
(TIF)

Acknowledgments

We are grateful to Claudio Caccia for help with analysis of sleep spindles, and to Anna Garofoli and Susanna Bianchi for technical assistance. We also acknowledge the valuable help of Roman S. Polishchuk of the Telethon Microscopy and Bio-Imaging Facility (Consorzio Mario Negri Sud) for immunogold staining of cultured neurons. This work is dedicated to the memory of Nicholas K. Gonatas (March 15, 1930, Thessaloniki, Greece—October 7, 2014, Philadelphia, PA, USA), luminary neuropathologist and dear friend.

Author Contributions

Conceived and designed the experiments: JC FT LI RC. Performed the experiments: IBo SM FDG IBe ER LC LT FB NFB MM CB GVB AP CB EM FF. Analyzed the data: IBo SM FDG IBe ER LC LT FB NFB MM CB GVB AP CB EM GF JC FF FT LI RC. Wrote the paper: FT LI RC.

References

1. Collinge J. Prion diseases of humans and animals: their causes and molecular basis. Annu Rev Neurosci. 2001; 24:519–50. Epub 2001/04/03. doi: 10.1146/annurev.neuro.24.1.519 24/1/519 [pii]. PMID: 11283320
2. Watts JC, Balachandran A, Westaway D. The expanding universe of prion diseases. PLoS Pathog. 2006; 2(3):e26. Epub 2006/04/13. doi: 10.1371/journal.ppat.0020026. PMID: 16609731
3. Will RG. Acquired prion disease: iatrogenic CJD, variant CJD, kuru. Br Med Bull. 2003; 66:255–65. Epub 2003/10/03. PMID: 14522863
4. Brown P, Gibbs CJ Jr., Rodgers-Johnson P, Asher DM, Sulima MP, Bacote A, et al. Human spongiform encephalopathy: the National Institutes of Health series of 300 cases of experimentally transmitted disease. Ann Neurol. 1994; 35(5):513–29. Epub 1994/05/01. doi: 10.1002/ana.410350504. PMID: 8179297
5. Colby DW, Prusiner SB. De novo generation of prion strains. Nat Rev Microbiol. 2011; 9(11):771–7. Epub 2011/09/29. nrmicro2650 [pii]doi: 10.1038/nrmicro2650. PMID: 21947062
6. Prusiner SB. Novel proteinaceous infectious particles cause scrapie. Science. 1982; 216(4542):136–44. Epub 1982/04/09. PMID: 6801762
7. Mastrianni JA. The genetics of prion diseases. Genet Med. 2010; 12(4):187–95. Epub 2010/03/11. doi: 10.1097/GIM.0b013e3181cd7374. PMID: 20216075
8. Mead S, Reilly MM. A new prion disease: relationship with central and peripheral amyloidoses. Nat Rev Neurol. 2015. Epub 2015/01/28. nrneurol.2014.263 [pii]doi: 10.1038/nrneurol.2014.263.
9. Goldfarb LG, Petersen RB, Tabaton M, Brown P, LeBlanc AC, Montagna P, et al. Fatal familial insomnia and familial Creutzfeldt-Jakob disease: disease phenotype determined by a DNA polymorphism. Science. 1992; 258(5083):806–8. Epub 1992/10/30. PMID: 1439789
10. Riek R, Wider G, Billeter M, Hornemann S, Glockshuber R, Wuthrich K. Prion protein NMR structure and familial human spongiform encephalopathies. Proc Natl Acad Sci U S A. 1998; 95(20):11667–72. Epub 1998/09/30. PMID: 9751723

11. Apetri AC, Vanik DL, Surewicz WK. Polymorphism at residue 129 modulates the conformational conversion of the D178N variant of human prion protein 90–231. Biochemistry. 2005; 44(48):15880–8. Epub 2005/11/30. doi: 10.1021/bi051455+. PMID: 16313190

12. Lee S, Antony L, Hartmann R, Knaus KJ, Surewicz K, Surewicz WK, et al. Conformational diversity in prion protein variants influences intermolecular beta-sheet formation. EMBO J. 2010; 29(1):251–62. Epub 2009/11/21. emboj2009333 [pii] doi: 10.1038/emboj.2009.333. PMID: 19927125

13. Chiesa R, Piccardo P, Ghetti B, Harris DA. Neurological illness in transgenic mice expressing a prion protein with an insertional mutation. Neuron. 1998; 21(6):1339–51. Epub 1999/01/12. S0896-6273(00)80653-4 [pii]. PMID: 9883727

14. Dossena S, Imeri L, Mangieri M, Garofoli A, Ferrari L, Senatore A, et al. Mutant prion protein expression causes motor and memory deficits and abnormal sleep patterns in a transgenic mouse model. Neuron. 2008; 60(4):598–609. Epub 2008/11/29. S0896-6273(08)00755-1 [pii] doi: 10.1016/j.neuron.2008.09.008. PMID: 19038218

15. Senatore A, Colleoni S, Verderio C, Restelli E, Morini R, Condliffe SB, et al. Mutant PrP suppresses glutamatergic neurotransmission in cerebellar granule neurons by impairing membrane delivery of VGCC $\alpha_2\delta$-1 subunit. Neuron. 2012; 74(2):300–13. Epub 2012/05/01. S0896-6273(12)00235-8 [pii] doi: 10.1016/j.neuron.2012.02.027. PMID: 22542184

16. Monari L, Chen SG, Brown P, Parchi P, Petersen RB, Mikol J, et al. Fatal familial insomnia and familial Creutzfeldt-Jakob disease: different prion proteins determined by a DNA polymorphism. Proc Natl Acad Sci U S A. 1994; 91(7):2839–42. Epub 1994/03/29. PMID: 7908444

17. Borbely AA. A two process model of sleep regulation. Hum Neurobiol. 1982; 1(3):195–204. Epub 1982/01/01. PMID: 7185792

18. Amici R, Cerri M, Ocampo-Garces A, Baracchi F, Dentico D, Jones CA, et al. Cold exposure and sleep in the rat: REM sleep homeostasis and body size. Sleep. 2008; 31(5):708–15. Epub 2008/06/04. PMID: 18517040

19. Chiesa R, Drisaldi B, Quaglio E, Migheli A, Piccardo P, Ghetti B, et al. Accumulation of protease-resistant prion protein (PrP) and apoptosis of cerebellar granule cells in transgenic mice expressing a PrP insertional mutation. Proc Natl Acad Sci U S A. 2000; 97(10):5574–9. Epub 2000/05/11. 97/10/5574 [pii]. PMID: 10805813

20. Nazor KE, Kuhn F, Seward T, Green M, Zwald D, Purro M, et al. Immunodetection of disease-associated mutant PrP, which accelerates disease in GSS transgenic mice. EMBO J. 2005; 24(13):2472–80. Epub 2005/06/18. 7600717 [pii]doi: 10.1038/sj.emboj.7600717. PMID: 15962001

21. Torres JM, Castilla J, Pintado B, Gutierrez-Adan A, Andreoletti O, Aguilar-Calvo P, et al. Spontaneous generation of infectious prion disease in transgenic mice. Emerg Infect Dis. 2013; 19(12):1938–47. Epub 2013/11/28. doi: 10.3201/eid1912.130106. PMID: 24274622

22. Tateishi J, Brown P, Kitamoto T, Hoque ZM, Roos R, Wollman R, et al. First experimental transmission of fatal familial insomnia. Nature. 1995; 376(6539):434–5. Epub 1995/08/03. doi: 10.1038/376434a0. PMID: 7630420

23. Brown P, Goldfarb LG, Kovanen J, Haltia M, Cathala F, Sulima M, et al. Phenotypic characteristics of familial Creutzfeldt-Jakob disease associated with the codon 178Asn *PRNP* mutation. Ann Neurol. 1992; 31(3):282–5. Epub 1992/03/11. doi: 10.1002/ana.410310309. PMID: 1353342

24. Telling GC, Parchi P, DeArmond SJ, Cortelli P, Montagna P, Gabizon R, et al. Evidence for the conformation of the pathologic isoform of the prion protein enciphering and propagating prion diversity. Science. 1996; 274(5295):2079–82. Epub 1996/12/20. PMID: 8953038

25. Fischer M, Rulicke T, Raeber A, Sailer A, Moser M, Oesch B, et al. Prion protein (PrP) with amino-proximal deletions restoring susceptibility of PrP knockout mice to scrapie. EMBO J. 1996; 15(6):1255–64. Epub 1996/03/15. PMID: 8635458

26. Chiesa R, Piccardo P, Quaglio E, Drisaldi B, Si-Hoe SL, Takao M, et al. Molecular distinction between pathogenic and infectious properties of the prion protein. J Virol. 2003; 77(13):7611–22. Epub 2003/06/14. PMID: 12805461

27. Zou WQ, Puoti G, Xiao X, Yuan J, Qing L, Cali I, et al. Variably protease-sensitive prionopathy: a new sporadic disease of the prion protein. Ann Neurol. 2010; 68(2):162–72. Epub 2010/08/10. doi: 10.1002/ana.22094. PMID: 20695009

28. Makarava N, Kovacs GG, Savtchenko R, Alexeeva I, Ostapchenko VG, Budka H, et al. A new mechanism for transmissible prion diseases. J Neurosci. 2012; 32(21):7345–55. Epub 2012/05/25. 32/21/7345 [pii]doi: 10.1523/JNEUROSCI.6351-11.2012. PMID: 22623680

29. Castilla J, Saa P, Hetz C, Soto C. In vitro generation of infectious scrapie prions. Cell. 2005; 121(2):195–206. Epub 2005/04/27. S0092-8674(05)00156-X [pii]doi: 10.1016/j.cell.2005.02.011. PMID: 15851027

30. Prusiner SB, Scott M, Foster D, Pan KM, Groth D, Mirenda C, et al. Transgenetic studies implicate interactions between homologous PrP isoforms in scrapie prion replication. Cell. 1990; 63(4):673–86. Epub 1990/11/16. 0092-8674(90)90134-Z [pii]. PMID: 1977523

31. Hill AF, Joiner S, Linehan J, Desbruslais M, Lantos PL, Collinge J. Species-barrier-independent prion replication in apparently resistant species. Proc Natl Acad Sci U S A. 2000; 97(18):10248–53. PMID: 10963685

32. Montagna P, Gambetti P, Cortelli P, Lugaresi E. Familial and sporadic fatal insomnia. Lancet Neurol. 2003; 2(3):167–76. Epub 2003/07/10. S1474442203003235 [pii]. PMID: 12849238

33. Sforza E, Montagna P, Tinuper P, Cortelli P, Avoni P, Ferrillo F, et al. Sleep-wake cycle abnormalities in fatal familial insomnia. Evidence of the role of the thalamus in sleep regulation. Electroencephalogr Clin Neurophysiol. 1995; 94(6):398–405. Epub 1995/06/01. 001346949400318F [pii]. PMID: 7607093

34. Tinuper P, Montagna P, Medori R, Cortelli P, Zucconi M, Baruzzi A, et al. The thalamus participates in the regulation of the sleep-waking cycle. A clinico-pathological study in fatal familial thalamic degeneration. Electroencephalogr Clin Neurophysiol. 1989; 73(2):117–23. Epub 1989/08/01. PMID: 2473878

35. Tobler I, Gaus SE, Deboer T, Achermann P, Fischer M, Rulicke T, et al. Altered circadian activity rhythms and sleep in mice devoid of prion protein. Nature. 1996; 380(6575):639–42. Epub 1996/04/18. doi: 10.1038/380639a0. PMID: 8602267

36. Manetto V, Medori R, Cortelli P, Montagna P, Tinuper P, Baruzzi A, et al. Fatal familial insomnia: clinical and pathologic study of five new cases. Neurology. 1992; 42(2):312–9. Epub 1992/02/01. PMID: 1736158

37. Taniwaki Y, Hara H, Doh-Ura K, Murakami I, Tashiro H, Yamasaki T, et al. Familial Creutzfeldt-Jakob disease with D178N-129M mutation of PRNP presenting as cerebellar ataxia without insomnia. J Neurol Neurosurg Psychiatry. 2000; 68(3):388. Epub 2000/04/29. PMID: 10787306

38. McLean CA, Storey E, Gardner RJ, Tannenberg AE, Cervenakova L, Brown P. The D178N (cis-129M) "fatal familial insomnia" mutation associated with diverse clinicopathologic phenotypes in an Australian kindred. Neurology. 1997; 49(2):552–8. Epub 1997/08/01. PMID: 9270595

39. Zerr I, Giese A, Windl O, Kropp S, Schulz-Schaeffer W, Riedemann C, et al. Phenotypic variability in fatal familial insomnia (D178N-129M) genotype. Neurology. 1998; 51(5):1398–405. Epub 1998/11/18. PMID: 9818868

40. Almer G, Hainfellner JA, Brucke T, Jellinger K, Kleinert R, Bayer G, et al. Fatal familial insomnia: a new Austrian family. Brain. 1999; 122 (Pt 1):5–16. Epub 1999/03/02. PMID: 10050890

41. Jackson WS, Borkowski AW, Faas H, Steele AD, King OD, Watson N, et al. Spontaneous generation of prion infectivity in fatal familial insomnia knockin mice. Neuron. 2009; 63(4):438–50. Epub 2009/08/28. S0896-6273(09)00581-9 [pii]doi: 10.1016/j.neuron.2009.07.026. PMID: 19709627

42. Chiesa R, Piccardo P, Biasini E, Ghetti B, Harris DA. Aggregated, wild-type prion protein causes neurological dysfunction and synaptic abnormalities. J Neurosci. 2008; 28(49):13258–67. Epub 2008/12/05. 28/49/13258 [pii]doi: 10.1523/JNEUROSCI.3109-08.2008. PMID: 19052217

43. Friedman-Levi Y, Mizrahi M, Frid K, Binyamin O, Gabizon R. PrP(ST), a soluble, protease resistant and truncated PrP form features in the pathogenesis of a genetic prion disease. PLoS One. 2013; 8(7): e69583. Epub 2013/08/08. doi: 10.1371/journal.pone.0069583PONE-D-13-18821 [pii]. PMID: 23922744

44. Collinge J, Whittington MA, Sidle KC, Smith CJ, Palmer MS, Clarke AR, et al. Prion protein is necessary for normal synaptic function. Nature. 1994; 370(6487):295–7. Epub 1994/07/28. doi: 10.1038/370295a0. PMID: 8035877

45. Krasnianski A, Bartl M, Sanchez Juan PJ, Heinemann U, Meissner B, Varges D, et al. Fatal familial insomnia: clinical features and early identification. Ann Neurol. 2008; 63(5):658–61. Epub 2008/03/25. doi: 10.1002/ana.21358. PMID: 18360821

46. Macchi G, Rossi G, Abbamondi AL, Giaccone G, Mancia D, Tagliavini F, et al. Diffuse thalamic degeneration in fatal familial insomnia. A morphometric study. Brain Res. 1997; 771(1):154–8. Epub 1998/02/12. S0006-8993(97)00902-5 [pii]. PMID: 9383019

47. Parchi P, Castellani R, Cortelli P, Montagna P, Chen SG, Petersen RB, et al. Regional distribution of protease-resistant prion protein in fatal familial insomnia. Ann Neurol. 1995; 38(1):21–9. Epub 1995/07/01. doi: 10.1002/ana.410380107. PMID: 7611720

48. Biasini E, Seegulam ME, Patti BN, Solforosi L, Medrano AZ, Christensen HM, et al. Non-infectious aggregates of the prion protein react with several PrPSc-directed antibodies. J Neurochem. 2008; 105 (6):2190–204. Epub 2008/02/27. JNC5306 [pii]doi: 10.1111/j.1471-4159.2008.05306.x. PMID: 18298665

49. Tapella L, Stravalaci M, Bastone A, Biasini E, Gobbi M, Chiesa R. Epitope scanning indicates structural differences in brain-derived monomeric and aggregated mutant prion proteins related to genetic prion

diseases. Biochem J. 2013; 454(3):417–25. Epub 2013/07/03. BJ20130563 [pii] doi: 10.1042/BJ20130563. PMID: 23808898

50. Sigurdson CJ, Nilsson KP, Hornemann S, Heikenwalder M, Manco G, Schwarz P, et al. De novo generation of a transmissible spongiform encephalopathy by mouse transgenesis. Proc Natl Acad Sci U S A. 2009; 106(1):304–9. Epub 2008/12/17. 0810680105 [pii]doi: 10.1073/pnas.0810680105. PMID: 19073920

51. Stohr J, Watts JC, Legname G, Oehler A, Lemus A, Nguyen HO, et al. Spontaneous generation of anchorless prions in transgenic mice. Proc Natl Acad Sci U S A. 2011; 108(52):21223–8. Epub 2011/12/14. 1117827108 [pii]doi: 10.1073/pnas.1117827108. PMID: 22160704

52. Friedman-Levi Y, Meiner Z, Canello T, Frid K, Kovacs GG, Budka H, et al. Fatal prion disease in a mouse model of genetic E200K Creutzfeldt-Jakob disease. PLoS Pathog. 2011; 7(11):e1002350. Epub 2011/11/11. doi: 10.1371/journal.ppat.1002350 PPATHOGENS-D-11-01077 [pii]. PMID: 22072968

53. Watts JC, Giles K, Stohr J, Oehler A, Bhardwaj S, Grillo SK, et al. Spontaneous generation of rapidly transmissible prions in transgenic mice expressing wild-type bank vole prion protein. Proc Natl Acad Sci U S A. 2012; 109(9):3498–503. Epub 2012/02/15. 1121556109 [pii] doi: 10.1073/pnas.1121556109. PMID: 22331873

54. Manson JC, Jamieson E, Baybutt H, Tuzi NL, Barron R, McConnell I, et al. A single amino acid alteration (101L) introduced into murine PrP dramatically alters incubation time of transmissible spongiform encephalopathy. EMBO J. 1999; 18(23):6855–64. Epub 1999/12/03. doi: 10.1093/emboj/18.23.6855. PMID: 10581259

55. Telling GC, Scott M, Mastrianni J, Gabizon R, Torchia M, Cohen FE, et al. Prion propagation in mice expressing human and chimeric PrP transgenes implicates the interaction of cellular PrP with another protein. Cell. 1995; 83(1):79–90. Epub 1995/10/06. 0092-8674(95)90236-8 [pii]. PMID: 7553876

56. Deleault NR, Piro JR, Walsh DJ, Wang F, Ma J, Geoghegan JC, et al. Isolation of phosphatidylethanolamine as a solitary cofactor for prion formation in the absence of nucleic acids. Proc Natl Acad Sci U S A. 2012; 109(22):8546–51. Epub 2012/05/16. 1204498109 [pii] doi: 10.1073/pnas.1204498109. PMID: 22586108

57. Biasini E, Medrano AZ, Thellung S, Chiesa R, Harris DA. Multiple biochemical similarities between infectious and non-infectious aggregates of a prion protein carrying an octapeptide insertion. J Neurochem. 2008; 104(5):1293–308. Epub 2007/11/24. JNC5082 [pii] doi: 10.1111/j.1471-4159.2007.05082.x. PMID: 18034781

58. Bessen RA, Kocisko DA, Raymond GJ, Nandan S, Lansbury PT, Caughey B. Non-genetic propagation of strain-specific properties of scrapie prion protein. Nature. 1995; 375(6533):698–700. Epub 1995/06/22. doi: 10.1038/375698a0. PMID: 7791905

59. Yang W, Cook J, Rassbach B, Lemus A, DeArmond SJ, Mastrianni JA. A new transgenic mouse model of Gerstmann-Sträussler-Scheinker syndrome caused by the A117V mutation of *PRNP*. J Neurosci. 2009; 29(32):10072–80. Epub 2009/08/14. 29/32/10072 [pii] doi: 10.1523/JNEUROSCI.2542-09.2009. PMID: 19675240

60. Chiesa R, Harris DA. Prion diseases: what is the neurotoxic molecule? Neurobiol Dis. 2001; 8(5):743–63. Epub 2001/10/11. doi: 10.1006/nbdi.2001.0433 S0969-9961(01)90433-4 [pii]. PMID: 11592845

61. Chiesa R. The elusive role of the prion protein and the mechanism of toxicity in prion disease. PLoS Pathog. 2015; In press.

62. Jucker M, Walker LC. Self-propagation of pathogenic protein aggregates in neurodegenerative diseases. Nature. 2013; 501(7465):45–51. Epub 2013/09/06. nature12481 [pii] doi: 10.1038/nature12481. PMID: 24005412

63. Mallucci G. Spreading proteins in neurodegeneration: where do they take us? Brain. 2013; 136(Pt 4):994–5. Epub 2013/03/23. awt072 [pii] doi: 10.1093/brain/awt072. PMID: 23518708

64. Drisaldi B, Stewart RS, Adles C, Stewart LR, Quaglio E, Biasini E, et al. Mutant PrP is delayed in its exit from the endoplasmic reticulum, but neither wild-type nor mutant PrP undergoes retrotranslocation prior to proteasomal degradation. J Biol Chem. 2003; 278(24):21732–43. Epub 2003/03/29. doi: 10.1074/jbc.M213247200 M213247200 [pii]. PMID: 12663673

65. Fioriti L, Dossena S, Stewart LR, Stewart RS, Harris DA, Forloni G, et al. Cytosolic prion protein (PrP) is not toxic in N2a cells and primary neurons expressing pathogenic PrP mutations. J Biol Chem. 2005; 280(12):11320–8. Epub 2005/01/06. M412441200 [pii] doi: 10.1074/jbc.M412441200. PMID: 15632159

66. Quaglio E, Restelli E, Garofoli A, Dossena S, De Luigi A, Tagliavacca L, et al. Expression of mutant or cytosolic PrP in transgenic mice and cells is not associated with endoplasmic reticulum stress or proteasome dysfunction. PLoS One. 2011; 6(4):e19339. Epub 2011/05/12. doi: 10.1371/journal.pone.0019339 PONE-D-11-01260 [pii]. PMID: 21559407

67. Massignan T, Biasini E, Lauranzano E, Veglianese P, Pignataro M, Fioriti L, et al. Mutant prion protein expression is associated with an alteration of the Rab GDP dissociation inhibitor alpha (GDI)/Rab11 pathway. Mol Cell Proteomics. 2010; 9(4):611–22. Epub 2009/12/10. M900271-MCP200 [pii] doi: 10.1074/mcp.M900271-MCP200. PMID: 19996123

68. Daude N, Lehmann S, Harris DA. Identification of intermediate steps in the conversion of a mutant prion protein to a scrapie-like form in cultured cells. J Biol Chem. 1997; 272(17):11604–12. Epub 1997/04/25. PMID: 9111077

69. Biasini E, Tapella L, Restelli E, Pozzoli M, Massignan T, Chiesa R. The hydrophobic core region governs mutant prion protein aggregation and intracellular retention. Biochem J. 2010; 430(3):477–86. Epub 2010/07/16. BJ20100615 [pii] doi: 10.1042/BJ20100615. PMID: 20626348

70. Senatore A, Restelli E, Chiesa R. Synaptic dysfunction in prion diseases: a trafficking problem? Int J Cell Biol. 2013; 2013:543803. Epub 2013/12/27. doi: 10.1155/2013/543803. PMID: 24369467

71. Khosravani H, Zhang Y, Tsutsui S, Hameed S, Altier C, Hamid J, et al. Prion protein attenuates excitotoxicity by inhibiting NMDA receptors. J Cell Biol. 2008; 181(3):551–65. Epub 2008/04/30. jcb.200711002 [pii] doi: 10.1083/jcb.200711002. PMID: 18443219

72. You H, Tsutsui S, Hameed S, Kannanayakal TJ, Chen L, Xia P, et al. Aβ neurotoxicity depends on interactions between copper ions, prion protein, and N-methyl-D-aspartate receptors. Proc Natl Acad Sci U S A. 2012; 109(5):1737–42. Epub 2012/02/07. 1110789109 [pii] doi: 10.1073/pnas.1110789109. PMID: 22307640

73. Watt NT, Taylor DR, Kerrigan TL, Griffiths HH, Rushworth JV, Whitehouse IJ, et al. Prion protein facilitates uptake of zinc into neuronal cells. Nat Commun. 2012; 3:1134. Epub 2012/10/18. ncomms2135 [pii] doi: 10.1038/ncomms2135. PMID: 23072804

74. Shepherd JD, Huganir RL. The cell biology of synaptic plasticity: AMPA receptor trafficking. Annu Rev Cell Dev Biol. 2007; 23:613–43. Epub 2007/05/18. doi: 10.1146/annurev.cellbio.23.090506.123516. PMID: 17506699

75. Traynelis SF, Wollmuth LP, McBain CJ, Menniti FS, Vance KM, Ogden KK, et al. Glutamate receptor ion channels: structure, regulation, and function. Pharmacol Rev. 2010; 62(3):405–96. Epub 2010/08/19. 62/3/405 [pii] doi: 10.1124/pr.109.002451. PMID: 20716669

76. Borchelt DR, Davis J, Fischer M, Lee MK, Slunt HH, Ratovitsky T, et al. A vector for expressing foreign genes in the brains and hearts of transgenic mice. Genet Anal. 1996; 13(6):159–63. Epub 1996/12/01. PMID: 9117892

77. Bueler H, Fischer M, Lang Y, Bluethmann H, Lipp HP, DeArmond SJ, et al. Normal development and behaviour of mice lacking the neuronal cell-surface PrP protein. Nature. 1992; 356(6370):577–82. Epub 1992/04/16. doi: 10.1038/356577a0. PMID: 1373228

78. Baracchi F, Opp MR. Sleep-wake behavior and responses to sleep deprivation of mice lacking both interleukin-1 beta receptor 1 and tumor necrosis factor-alpha receptor 1. Brain Behav Immun. 2008; 22(6):982–93. Epub 2008/03/11. S0889-1591(08)00040-8 [pii] doi: 10.1016/j.bbi.2008.02.001. PMID: 18329246

79. Schindelin J, Arganda-Carreras I, Frise E, Kaynig V, Longair M, Pietzsch T, et al. Fiji: an open-source platform for biological-image analysis. Nat Methods. 2012; 9(7):676–82. Epub 2012/06/30. nmeth.2019 [pii] doi: 10.1038/nmeth.2019. PMID: 22743772

80. Giaccone G, Canciani B, Puoti G, Rossi G, Goffredo D, Iussich S, et al. Creutzfeldt-Jakob disease: Carnoy's fixative improves the immunohistochemistry of the proteinase K-resistant prion protein. Brain Pathol. 2000; 10(1):31–7. Epub 2000/02/11. PMID: 10668893

81. Beznoussenko GV, Dolgikh VV, Seliverstova EV, Semenov PB, Tokarev YS, Trucco A, et al. Analogs of the Golgi complex in microsporidia: structure and avesicular mechanisms of function. J Cell Sci. 2007; 120(Pt 7):1288–98. Epub 2007/03/16. jcs.03402 [pii] doi: 10.1242/jcs.03402. PMID: 17356068

82. Saa P, Castilla J, Soto C. Cyclic amplification of protein misfolding and aggregation. Methods Mol Biol. 2005; 299:53–65. Epub 2005/06/28. 1-59259-874-9:053 [pii]. PMID: 15980595
